# Activity-Dependent Gating of Calcium Spikes by A-type K+ Channels Controls Climbing Fiber Signaling in Purkinje Cell Dendrites

**DOI:** 10.1016/j.neuron.2014.08.035

**Published:** 2014-10-01

**Authors:** Yo Otsu, Païkan Marcaggi, Anne Feltz, Philippe Isope, Mihaly Kollo, Zoltan Nusser, Benjamin Mathieu, Masanobu Kano, Mika Tsujita, Kenji Sakimura, Stéphane Dieudonné

**Affiliations:** 1 Inhibitory Transmission Team, IBENS, CNRS UMR UMR8197, INSERM U1024, Ecole Normale Supérieure, 75005 Paris, France; 2 Cerebellum Group, IBENS, CNRS UMR UMR8197, INSERM U1024, Ecole Normale Supérieure, 75005 Paris, France; 3 Institut des Neurosciences Cellulaires et Intégratives, CNRS UPR 3212, 67000-Strasbourg, France; 4 Laboratory of Cellular Neurophysiology, Institute of Experimental Medicine of the Hungarian Academy of Sciences, 1083 Budapest, Hungary; 5 Imaging Facility, IBENS, CNRS UMR 8197, INSERM U1024, Ecole Normale Supérieure, 75005 Paris, France; 6 Department of Neurophysiology, Graduate School of Medicine, University of Tokyo, Tokyo 113-0033, Japan; 7 Center for Transdisciplinary Research, Niigata University, Niigata 950-2181, Japan; 8 Department of Cellular Neurobiology, Brain Research Institute, Niigata University, Niigata 951-8585, Japan

## Abstract

In cerebellar Purkinje cell dendrites, heterosynaptic calcium signaling induced by the proximal climbing fiber (CF) input controls plasticity at distal parallel fiber (PF) synapses. The substrate and regulation of this long-range dendritic calcium signaling are poorly understood. Using high-speed calcium imaging, we examine the role of active dendritic conductances. Under basal conditions, CF stimulation evokes T-type calcium signaling displaying sharp proximodistal decrement. Combined mGluR1 receptor activation and depolarization, two activity-dependent signals, unlock P/Q calcium spikes initiation and propagation, mediating efficient CF signaling at distal sites. These spikes are initiated in proximal smooth dendrites, independently from somatic sodium action potentials, and evoke high-frequency bursts of all-or-none fast-rising calcium transients in PF spines. Gradual calcium spike burst unlocking arises from increasing inactivation of mGluR1-modulated low-threshold A-type potassium channels located in distal dendrites. Evidence for graded activity-dependent CF calcium signaling at PF synapses refines current views on cerebellar supervised learning rules.

## Highlights

• The giant proximal CF input controls Ca^2+^ plasticity at distal PF-Purkinje synapses
• We find mGluR1 activity and Purkinje cell depolarization to control CF calcium influx
• Distal CF signaling is mediated by centrifugal dendritic calcium spikes bursts
• Dendritic low-threshold A-type K+ channels modulation underlie calcium spike gating

## Teaser

Climbing fibers supervise cerebellar learning expressed as calcium-dependent plasticity at the parallel synapses on Purkinje cells. Otsu et al. find that activity-dependent parameters control heterosynaptic climbing fiber signaling by gating calcium spikes in Purkinje cell dendrites.

## Introduction

Interactions between synaptic inputs, dendritic excitability, and dendritic morphology give rise to local and global calcium signaling in dendrites (Higley and Sabatini, 2008, Larkum et al., 1999, Sjöström et al., 2008). These interactions shape the rules for the induction of calcium-dependent plasticity and ultimately control information processing and storage in neuronal networks (Magee and Johnston, 2005, Sjöström et al., 2008).

Climbing fibers (CFs) form a giant synaptic input on spines on large-diameter proximal dendrites of cerebellar Purkinje cells and control calcium dependent short- and long-term plasticity at parallel fiber (PF) synapses on spiny dendritic branchlets (Brenowitz and Regehr, 2005, Rancz and Häusser, 2006, Wang et al., 2000), the main site for cerebellar learning. It is crucial to understand the conditions under which heterosynaptic modifications of PF inputs occur, and therefore the nature and regulation of dendritic CF calcium signaling. CF stimulations evoke widespread calcium transients in Purkinje cell dendrites (Sullivan et al., 2005, Tank et al., 1988), which have been attributed to propagating dendritic calcium spikes. While regenerative events have been recorded from proximal smooth dendrites both in vivo (Fujita, 1968, Kitamura and Häusser, 2011) and in vitro (Davie et al., 2008, Llinás and Sugimori, 1980), the variability of CF calcium transients measured in distal spiny branchlets suggests that calcium spikes may not always occur at distal sites. The amplitude of the CF calcium signal is modulated by the somatic holding potential (Wang et al., 2000, Kitamura and Häusser, 2011), by dendritic field depolarization (Midtgaard et al., 1993), by synaptic inhibition of the dendrites (Callaway et al., 1995, Kitamura and Häusser, 2011), and by the activity of PFs (Brenowitz and Regehr, 2005, Wang et al., 2000). The mechanisms underlying these modulations remain unknown.

Purkinje cells express a high density of P/Q-type (Usowicz et al., 1992) and T-type (Hildebrand et al., 2009) calcium channels. P/Q-type channels sustain propagating high-threshold dendritic calcium spikes (Fujita, 1968, Llinás et al., 1968, Llinás and Sugimori, 1980). In contrast, T-type channels are involved in local spine-specific calcium influx during PF bursts (Hildebrand et al., 2009). Purkinje cell dendrites also express a variety of voltage-gated potassium channels, but their roles in the regulation of dendritic calcium electrogenesis are poorly understood (Etzion and Grossman, 1998, Llinás and Sugimori, 1980, McKay and Turner, 2004, Womack and Khodakhah, 2004). Here, we used random-access multiphoton (RAMP) microscopy to monitor the calcium transients induced by CF stimulation (CF-evoked calcium transients [CFCTs]) at high temporal resolution to unambiguously distinguish between subthreshold calcium transients and calcium spikes. We show that calcium spike initiation and propagation in distal spiny branchlets are controlled by activity-dependent mechanisms.

## Results

### Proximodistal Decrement of CFCTs in Purkinje Cell Dendrites

**Figure 1:**
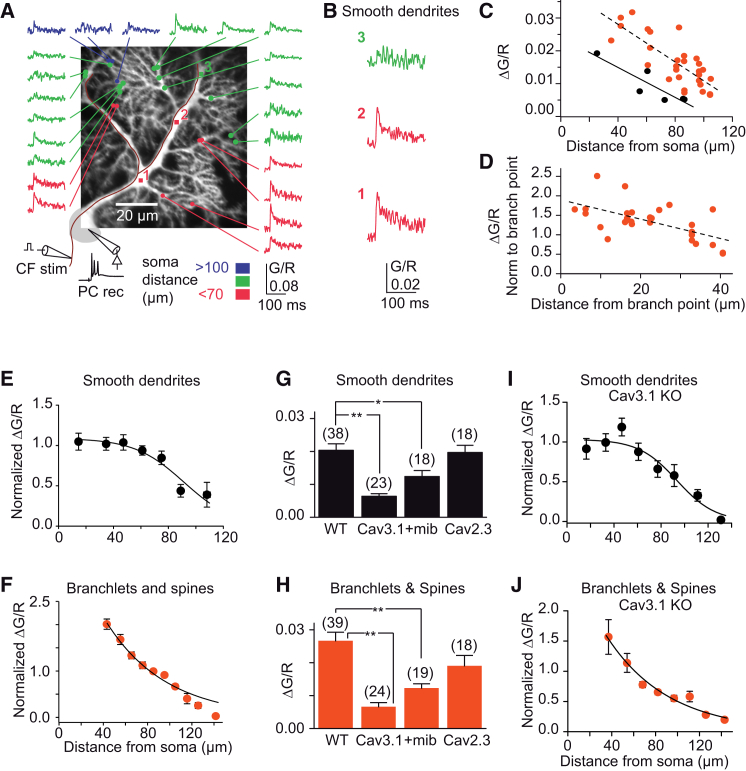
Spatial Decrement of Calcium Transients Evoked by Complex Spikes (A) Quasisimultaneous recordings of fluorescence transients in response to CF stimuli in 22 spines and dendritic shafts of a Purkinje cell loaded with 200 μM Fluo-4. Fluo-4 fluorescence is normalized to the calcium-independent Alexa 594 fluorescence (G/R) and optical traces represent the average of 28 stimuli. Each CF stimulation induced an all-or-none complex spike at the soma. Recording site distance from soma is color coded. (B) Fluorescence transients in the main smooth dendrite at points marked in (A). (C) Relationship between calcium transient amplitude and distance from soma (same cell as in A). Black circles: smooth dendrites, plain regression line (slope 0.023 ± 0.006 ΔG/R per 100 μm [±SD]; p < 0.05). Red circles: spiny branchlets and spines, short dashes regression line (slope 0.028 ± 0.004 ΔG/R per 100 μm (±SD); p < 0.001). (D) Same as (C) for the distance of recording site in spiny branchlets and spines from the branch point in smooth dendrite. Signal is normalized to the signal in smooth dendrite at the branch point. The intercept of dashed regression line (slope 0.025 ± 0.007 μm^−1^ (±SD); p < 0.001) is 1.9: this higher calcium concentration at the base of the spiny branchlet than in the smooth dendrite is likely due to the higher surface-to-volume ratio of spiny branchlets. (E and F) Spatial pattern of CFCTs from WT mice. Calcium transients were normalized to the value of ΔG/R in the proximal smooth dendrites and plotted against the soma distance. Points are average of 6–49 values per 15 μm (13 cells), and continuous lines show logistic function fit to smooth dendrites data (E) and a single exponential fit to pooled spiny branchlets and spines data (F). (G and H) Pharmacogenetic profile of CFCTs measured from slices of WT, Cav3.1 and Cav2.3 KO mice, and from mibefradil-treated WT slices. In each cell, more than 20 CF stimuli were averaged. Cell number is indicated on each bar. Error bars show ±SEM. ^∗∗^p < 0.01, ^∗^p < 0.05. For Cav2.3, p = 0.86 in smooth dendrites (G), n = 18 cells, p = 0.13 in spines and spiny branchlets (H). (I and J) As in (E) and (F), for CFCTs in CaV3.1 KO mice (4–131 values per 15μm; 16 cells). The small remaining CFCTs in CaV3.1 KOs also decrease with distance from soma.

CFCTs were mapped optically in Purkinje cell smooth and spiny dendrites using RAMP microscopy (Otsu et al., 2008). At repetition rates close to 1 kHz, the peak of Fluo-4 (200 μM) fluorescence transients was well resolved (Figure S1 available online). Using dual indicator quantitative measurements (see Experimental Procedures), we found that the amplitude of the CFCT (Figures 1A and 1B) decreased with distance from the soma (Figure 1C). In individual spiny dendrites, CFCT amplitude decreased linearly as a function of the distance from the parent dendritic trunk (Figure 1D) by −1.4% ± 0.4% μm^−1^ (±SD) for spines (r = −0.26, p < 0.001; n = 157 of 14 cells), and −1.5% ± 0.4% μm^−1^ for spiny branchlet shafts (r = −0.36, p < 0.001; n = 114 of 14 cells). In proximal compartments (<50 μm from soma), fluorescence transients averaged 0.023 ± 0.008 ΔG/R (±SD) in spines (n = 15, 5 cells), 0.020 ± 0.008 ΔG/R in spiny branchlets (n = 19, 7 cells), and 0.014 ± 0.008 ΔG/R in smooth dendrites (n = 25, 10 cells). In the most distal parts (>120 μm from soma), CFCTs were barely detectable (0.003 ± 0.004 ΔG/R [±SD] in spines, n = 22, 4 cells; 0.002 ± 0.002 ΔG/R in spiny branchlets, n = 18, 4 cells).

The average spatial profile of the CFCT was obtained by pooling data from 13 cells. In the smooth dendrites, the CFCT remained constant up to ∼70 μm from the soma and decreased markedly in more distal parts (Figure 1E). Half-maximum occurred at 91 μm from the soma with a steepness of 18 μm (exponential space constant of the logistic fit). In contrast, the amplitude of the CFCTs in spiny branchlets and in spines decreased approximately exponentially with distance from the soma (space constant; λ = 54.5 μm) (Figure 1F). This spatial profile of calcium influx is reminiscent of the electrotonic distribution of membrane potentials in Purkinje cells upon proximal depolarization (Roth and Häusser, 2001), suggesting that calcium transients result from electrotonic activation of calcium channels in spiny dendrites.

### Low-Threshold Calcium Channels Mediate Decremental CFCTs

In Purkinje cells of Cav3.1 knockout (KO) mice, lacking the main T-type subunit, the amplitude of the CFCTs was reduced to 31% of wild-type (WT) mice (n = 23 cells, p < 0.001) in smooth dendrites and to 25% of WT (n = 24 cell, p < 0.001) in spines and spiny branchlets (Figures 1G and 1H). In contrast, the CFCTs were not significantly inhibited in Cav2.3 KO mice lacking R-type calcium channels (Figures 1G and 1H). The role of Cav3 channels was confirmed by pharmacological block with 1 μM mibefradil (McDonough and Bean, 1998), which reduced the CFCTs to 61% (p = 0.012) (Figure 1G) and to 46% (p < 0.001) of control in smooth dendrites and in spines and spiny branchlets (Figure 1H), respectively. The spatial profile of the CFCTs recorded from Cav3.1 KO mice was similar to that observed in WT mice, with a half decrement at 93.5 μm (steepness of 16.3 μm) in the smooth dendrites and a λ = 56.3 μm in the spiny dendrites (Figures 1I and 1J). In conclusion, electrotonic filtering of the CF excitatory postsynaptic potential (EPSP) in spiny branchlets reduces calcium signaling at distal PF synapses, which is mainly mediated by T-type channels.

### mGluR1 Activation Unlocks Dendritic Calcium Spikes and Enables Heterosynaptic CF Calcium Signaling

**Figure 2:**
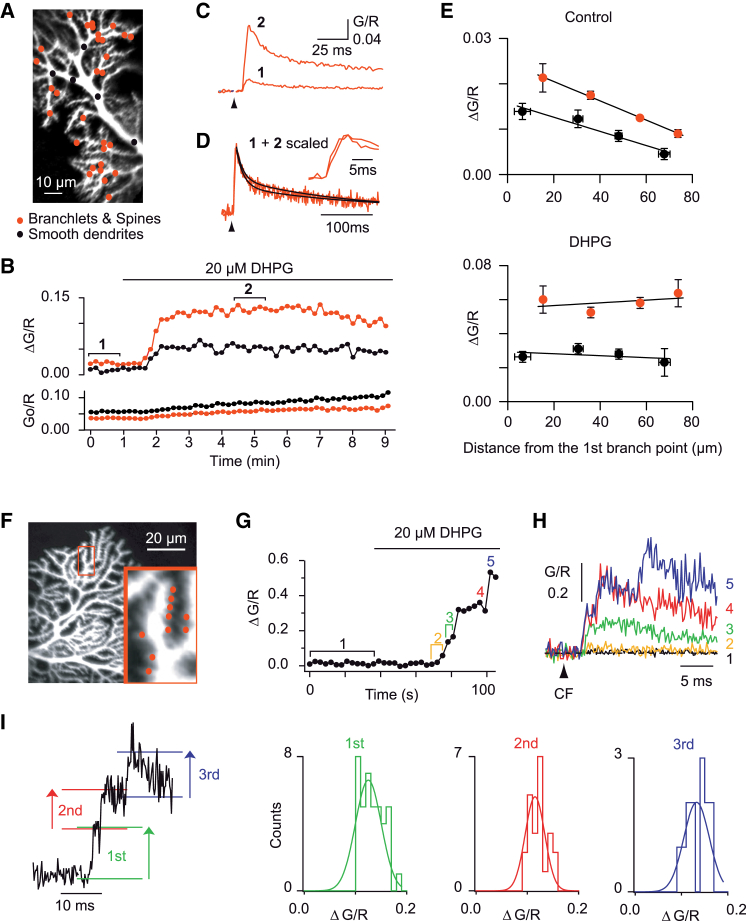
mGluR1 Activation Potentiates the CFCTs, which Are Then Mediated by Bursts of Propagating Calcium Spikes (A–E) Effect of mGluR1 activation in a cell loaded with 200 μM Fluo-4. (A) Morphological image of a Purkinje cell from a WT mouse showing the POIs from which measurements in (B)–(D) were obtained (black dots: smooth dendrite; red dots: spiny branchlets and spines). (B) CFCT (ΔG/R) potentiation and basal fluorescence (Go/R) increase upon bath application of 20 μM DHPG. Fluorescence transients averaged amplitude over all POIs in smooth dendrites (black circles) and spiny branchlets and spines (red circles). Each point represents a single CF stimulation at 0.1 Hz. (C) Control and potentiated CFCTs in spiny branchlets and spines averaged over six stimulations (1 min) at times (1) and (2), as indicated in (B). (D) Scaled control and potentiated CFCTs from (C), showing their similar onset and decay time course. (E) Effect of DHPG in smooth dendrites (black) and spiny branchlets and spines (red) depends on the distance to the first branch point in smooth dendrites (six cells). CFCTs in the POIs induced by 15–42 (control) and by 6–12 CF stimuli (DHPG) were pooled and averaged every 20–30 μm (7–78 POIs). For direct comparison, identical bins were used for control and in the presence of DHPG measurements. Linear regression lines are shown in control: smooth dendrites (−1.26% ± 0.24% μm^−1^ [±SD], r = −0.66, p < 0.0001; 39 POIs, 6 cells), spiny branchlets and spines (−1.03% ± 0.10% μm^−1^ [±SD], r = −0.59, p < 0.0001; 191 POIs, 6 cells), and after DHPG application: smooth dendrites (r = −0.15, p = 0.38; 39 POIs, 6 cells), spiny branchlets and spines (r = 0.06, p = 0.39; 191 POIs, 6 cells). (F–I) Recordings at higher time resolution in a cell loaded with 500 μM Fluo-5F reveals calcium spikes in distal Purkinje cell spines in presence of 20 μM DHPG. (F) Morphology of a Purkinje cell showing the distal location of the recorded spines. All ten POIs are placed on spines of two adjacent branchlets, as shown in inset (red dots) and characterized in (G)–(I). (G) Time course of the DHPG potentiation of CFCTs. Each point shows the amplitude of the fluorescence transient induced by a CF stimulation (0.33 Hz) and averaged over the ten POIs. (H) Fluorescence transients recorded at a repetition rate of 5 kHz at various time points during the onset of the DHPG effect (indicated by numbers in G). Traces are averages of 13 (at 1), 3 (2), 2 (3), or single (4, 5) CF stimuli. Note the multiphasic onset and stepwise amplitude increase of the CFCTs after DHPG application. (I) Example of a CFCT displaying three unitary transients induced by a single CF stimulation (left column). Amplitude histogram of the first, second, and third unitary transients in CFCTs obtained from the same cell. Gaussian curves are fitted to the data.

We explored whether PF input-mediated glutamatergic signaling might promote CF-evoked dendritic calcium electrogenesis. Selective mGluR1 activation by DHPG potentiated CFCTs by 350% ± 80% in spiny branchlets and by 320% ± 120% in smooth dendrites (n = 8 cells; paired data) (Figures 2A–2D). This effect developed in a few tens of seconds, as DHPG penetrated into the slice and was accompanied by a slower increase of basal calcium concentration (slope 4% ± 1%.min^−1^ [±SD]) (Figure 2B). The somatic complex spike remained unchanged (Figure S2), confirming that 20 μM DHPG did not depress the CF EPSP (Maejima et al., 2005). Strikingly, the potentiated CFCT no longer showed decrease with distance from the soma (Figure 2E), an effect that cannot be attributed to dye saturation (see Supplemental Information).

Does the potentiation of CF calcium signaling result from the occurrence of P/Q dendritic calcium spikes in distal dendrites? In small compartments, like spines, the rising phase of optical calcium transients monitored with high binding rate calcium dyes is expected to reflect the time course of the underlying calcium conductance (Cornelisse et al., 2007). Using 500 μM Fluo-5F, we performed optical recordings of the CFCTs at a frame rate of 4.8 kHz. The signal-to-noise ratio was preserved by pooling photons collected from ten POIs distributed over one or two adjacent spiny branchlets (Figure 2F). At this temporal resolution, mGluR1-potentiated CFCTs appeared as composite events made of several fast-rising unitary fluorescence transients (n = 17 of 18) (Figures 2G and 2H).

Unitary transients could be resolved without averaging and their number gradually increased as the mGluR1 potentiation developed (Figure 2H). The mean amplitude of unitary transients varied widely from cell to cell (first transient 0.102 ± 0.040 [±SD] ΔG/R, 343 events, 7 sites in 6 cells, p < 0.001; second transient 0.095 ± 0.039 ΔG/R, 201 events, 7 sites in 6 cells; 3rd transient 0.136 ± 0.040 ΔG/R, 32 events, 5 sites in 4 cells). However, in a given cell, the amplitude distribution of unitary transients was narrow (Figure 2I) and their mean amplitude was independent of their position in the global response (second over first 0.97 ± 0.02, p = 0.89; third over first 1.01 ± 0.04, p = 0.52). We conclude that all-or-none unitary transients are signatures of dendritic spikes.

### Purkinje Cell Depolarization Determines the Number of Dendritic Calcium Spikes in DHPG-Potentiated Composite CFCTs

**Figure 3:**
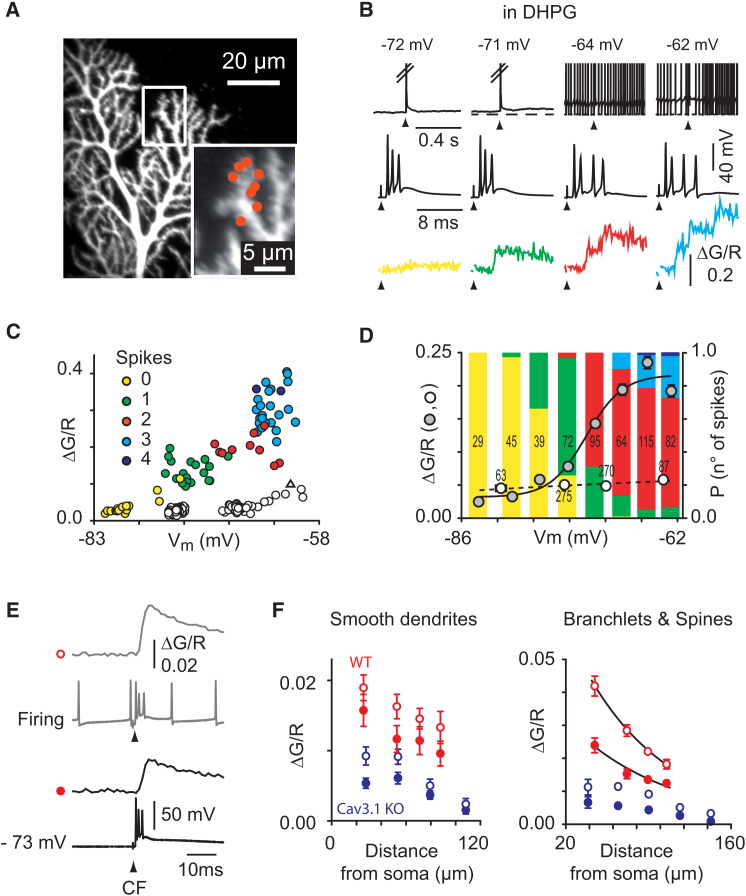
Comparison of Membrane Potential Effect on CFCTs in the Presence and the Absence of DHPG (A) DHPG was applied while recording CFCTs (500 μM Fluo-5F) in eight distally located spines of a Purkinje cell from a WT mouse (see red dots in inset). (B) Examples of the electrophysiological traces (top, and middle on expanded timescale) at different holding potentials when applying a CF stimulation (at arrowhead) in the same cell as in (A) and (C). Note that in top trace, spikes are cut. Corresponding illustrated CFCTs (bottom traces) are single stimulus traces (green, red, and blue) or average of three CF stimuli (yellow). The number of calcium spike is color coded. Broken line indicates −73 mV. (C) Relationship between composite CFCT amplitude, color-coded calcium spike number, and somatic membrane potential in this cell. Open circles show CFTC amplitude before DHPG application. A spike-like CFCT was recorded in one case (open triangle). (D) Group data from six cells. Color fillings in each bar indicate the probability of triggering a given number of spikes. Data were binned every 2–7 mV of somatic membrane potential. The number in each bar indicates the number of occurrences. ΔG/R values recorded after DHPG application (gray circles) are fitted with a logistic function. Open circles correspond to the CFCTs amplitude (no spike) recorded before DHPG application in the same cells. Error bars indicate ±SEM. (E) An example in a WT mouse Purkinje cell of CFCTs (200 μM Fluo-4) and somatic membrane potential recordings in response to CF stimuli at hyperpolarized (−73 mV imposed by somatic current injection, circle) and depolarized (let to fire spontaneously with 0 pA holding current, open circle) membrane potentials in the absence of DHPG. (F) Plot of CFCTs amplitudes at both membrane potentials as a function of the recording point distance from the soma. Note that the calcium signals are potentiated by somatic depolarization but still decay with distance from the soma. In red and blue circles, data obtained from WT (n = 6 cells) and Ca_V_3.1 KO (n = 11 cells) mice, respectively.

In the presence of DHPG, the number of unitary calcium transients (P/Q dendritic spikes) and the resulting peak amplitude of the composite CFCT were tightly correlated with the somatic membrane potential (Figures 3A–3D). While hyperpolarization caused dendritic calcium spike failure, gradual depolarization from −75 mV to −60 mV increased the number of dendritic calcium spikes in the CFCT (Figures 3A–3D). Overall, the number of dendritic calcium spikes and the CFCT amplitude were related to the membrane potential by a logistic sigmoidal relationship with a half-maximum of −72.3 mV and an exponential steepness of 2.0 mV (6 cells) (Figure 3D). In contrast, before addition of DHPG, the amplitude of the CFCT was only mildly increased by somatic depolarization (Figures 3C and 3D) and a fast-rising unitary calcium transient was only recorded in one trial at the most depolarized potentials (triangle in Figure 3C).

In control experiments without DHPG, Purkinje cells were either held around −70 mV or set to fire spontaneously (42.7 ± 4.2 Hz, n = 14; membrane potential: −62 ± 1.7 mV) and a spatial mapping of the CFCT was performed (Figures 3E and 3F). CFCTs were potentiated by depolarization to 143.8% ± 13% of control in smooth dendrites (n = 14, p = 0.002) and to 174.1% ± 19% of control in spiny branchlets (n = 14, p = 0.001) (Figure 3F). Depolarization did not reduce the spatial decrement of the CFCTs (linear regression slope −0.011 ± 0.007/μm [±SD] versus −0.010 ± 0.008/μm in smooth dendrites, 5 cells; λ = 47.4 μm versus 50.7 μm in spines and spiny branchlets, 6 cells) (Figure 3F). Furthermore, in Cav3.1 KO mice, the CFCTs were similarly reduced at hyperpolarized potentials or depolarized potentials (to 36.5% and 42.6% of WT, respectively, in smooth dendrites; to 28.2% and 34.4% of WT, respectively, in spiny dendrites). We conclude that mGluR1 activation is strictly required and acts in synergy with depolarization to unlock dendritic P/Q calcium spiking. This synergistic effect is not caused by direct mGluR1-mediated depolarization of the dendrites. Indeed, blockade by 1-naphthyl acetyl spermine (NASPM) of the slow current responsible for mGluR1 depolarization did not prevent unlocking (Supplemental Information and Figure S3).

### P/Q-type Calcium Channels Are the Main Contributors to CFCTs after DHPG Potentiation

**Figure 4:**
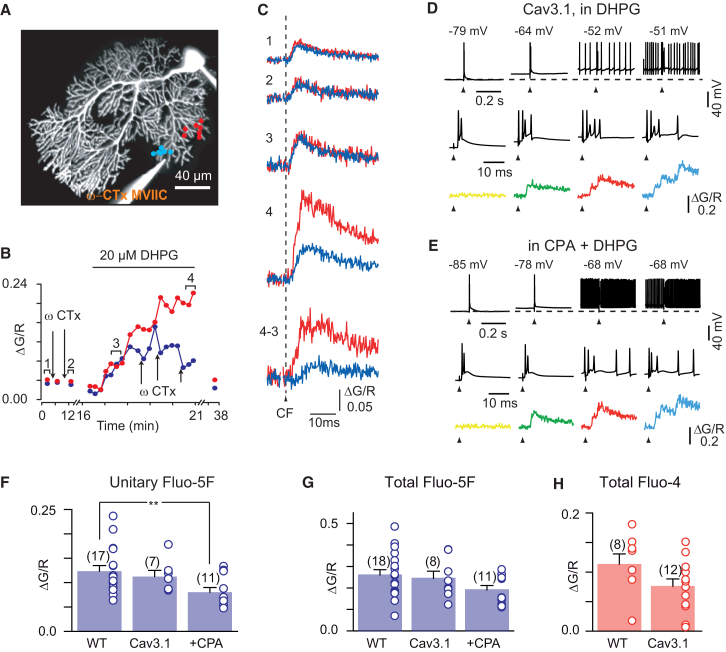
Pharmacology of the DHPG-Induced Ca Spike (A–C) Fast unitary transient induced by DHPG are blocked by ω-conotoxin MVIIC. (A) ωCTx was pressure applied to a small region of PC dendrites, using a nearby spiny branchlet further from the puff pipette as a control. Panel shows configuration with 14 optical recording sites and puff pipette. (B) Time course of the CFCT amplitude potentiation by DHPG for the POIs in (A) for test (in blue) and control (in red). Arrows point to puff applications of ωCTx. (C) Fluorescence transients recorded in each region at times indicated in (B). ωCTx puff did not significantly reduce the CFCTs at times 1 and 2. After ωCTx partial washout, DHPG application produced a subthreshold potentiation of the CFCTs in control and test regions with similar time course and amplitude (time 3). Repeated ωCTx puffs prevented the appearance of fast unitary transients at the test site, but not at the control site, and eventually reversed the CFCTs to the subthreshold slow-rising level (time 4). Overall ωCTx led to the suppression of fast unitary transients. (D and E) DHPG-induced CFCTs recorded in Ca_V_3.1 KO slices (D) and in cyclopiazonic acid-treated slices (CPA) of WT mice (E). As in Figure 3B, examples of the electrophysiological traces (top, and middle on expanded timescale) and corresponding illustrated CFCTs (bottom traces) when applying a CF stimulation (at arrow). Note evoked unitary events as in Figures 3A–3D. Broken lines indicate −78 mV (D) and −85 mV (E). (F–H) Pharmacogenetic profile of the CFCTs induced by DHPG in WT slices nontreated/treated with 25 μM cyclopiazonic acid (CPA) and in CaV3.1 KO slices. Amplitude of the first unitary transient (F) and total amplitude (G) of the CFCTs in presence of DHPG are reported. Each dot (average of 5–10 spines recordings) corresponds to a branchlet (500 μM Fluo-5). (H) Each dot corresponds to a cell as in Figure 1H (200 μM Fluo-4). During DHPG application, ten CF stimuli were averaged at the peak of the response. Numbers of cells or branchlets are indicated on each bar. Errors bars show ±SEM. ^∗∗^p < 0.01.

We applied ω-conotoxin MVIIC locally on a spiny branchlet and simultaneously monitored calcium at the application site and in a nearby control branchlet. In baseline conditions (without DHPG) ω-conotoxin MVIIC puff did not significantly reduce the CFCTs (Figures 4A–4C, time 1 and 2). In contrast, in DHPG, unitary transients were suppressed by ω-conotoxin MVIIC (Figures 4A–4C) at the application site but not in the control site, leaving an underlying low-amplitude slow-rising transient. Overall ω-conotoxin MVIIC inhibited suprathreshold CFCTs to 49.7% ± 10% of control regions in the same dendrite (n = 3) and suppressed all unitary transients. This further supports that unitary transients are the signature of high-threshold P/Q calcium spikes.

mGluR1 potentiation of T-type calcium channels at Purkinje cell spines has been recently reported (Hildebrand et al., 2009). T-type calcium channels may thus contribute to unitary transients by triggering P/Q spikes. However, unitary calcium transients were readily evoked in Cav3.1 KO mice (in the presence of DHPG), with similar voltage dependence as in WT mice (n = 7 out of 8) (Figure 4D) and similar amplitude (0.11 ± 0.01 ΔG/R in Cav3.1 KO, n = 7; 0.12 ± 0.01 ΔG/R in WT, n = 17; p = 0.71; Figure 4F). The maximum amplitude of the composite DHPG-potentiated CFCTs in spiny branchlets was mildly reduced in the Cav3.1 KO, when compared to WT (92% ± 14%; 0.24 ± 0.03 ΔG/R in Cav3.1 KO, n = 8; 0.26 ± 0.02 ΔG/R in WT, n = 18; p = 0.72 when measured with 500 μM Fluo-5F; Figure 4G) (68% ± 20%; 0.075 ± 0.01 ΔG/R in Cav3.1 KO, n = 12; 0.11 ± 0.02 ΔG/R in WT, n = 8; p = 0.076 when measured with 200 μM Fluo-4; Figure 4H). T-type channels may thus provide a contribution of about 20% (average reduction for the Fluo-4 and Fluo-5F conditions) to the total amplitude of mGluR1-potentiated CF calcium transients, similar to the amplitude of T-type mediated influx in control conditions.

Another possible source of cytoplasmic calcium linked to mGluR1 receptor activation is IP3-dependent calcium stores (Finch and Augustine, 1998, Takechi et al., 1998), as IP3 uncaging preceding the CF stimulations has been shown to produce a late component of the CFCT (Sarkisov and Wang, 2008). In our experiments, the time to peak of the CFCT was not significantly slowed by DHPG potentiation (increased delay to peak after DHPG: 0.94 ± 3.0 ms [±SD] in spines, 2.2 ± 4.4 ms [±SD] in spiny branchlets and 2.1 ± 1.5 ms [±SD] in smooth dendrites, n = 5 cells, p > 0.05) (Figure 2D), contrary to what has been observed to date for the slow secondary release of calcium from IP3-sensitive calcium stores (Finch and Augustine, 1998, Sarkisov and Wang, 2008, Takechi et al., 1998). Slices were preincubated with 25 μM cyclopiazonic acid (CPA), to empty the internal stores. In these conditions, DHPG strikingly potentiated the CFCTs by evoking unitary transients that were recruited in a voltage-dependent manner, as in control (n = 11 out of 11) (Figure 4E). Hence calcium stores, if recruited, act downstream of spike unlocking by mGluR1 activation. The mean amplitude of the unitary transients was reduced to 0.08 ± 0.01 ΔG/R (65% of control, n = 11; p = 0.008) and the total amplitude of the CFCT was reduced to 0.19 ± 0.02 ΔG/R (73% of control, n = 11; p = 0.068). Participation of IP3-dependent calcium stores in submillisecond calcium release (unitary transients) is unexpected, as all store release events described in Purkinje cells have an onset time course of several milliseconds (Finch and Augustine, 1998, Takechi et al., 1998) even when paired with CF stimulation (Sarkisov and Wang, 2008). Alternatively, nonspecific effects, as attested by significant slice swelling during CPA application, may explain the reduction in spike-associated calcium influx. Overall, our data demonstrate that unitary transients mediated by dendritic P/Q spike are the primary contributors to voltage-dependent CFCT potentiation by mGluR1 activation.

### Optical Dissection of Subthreshold and Suprathreshold Dendritic Calcium Electrogenesis

**Figure 5:**
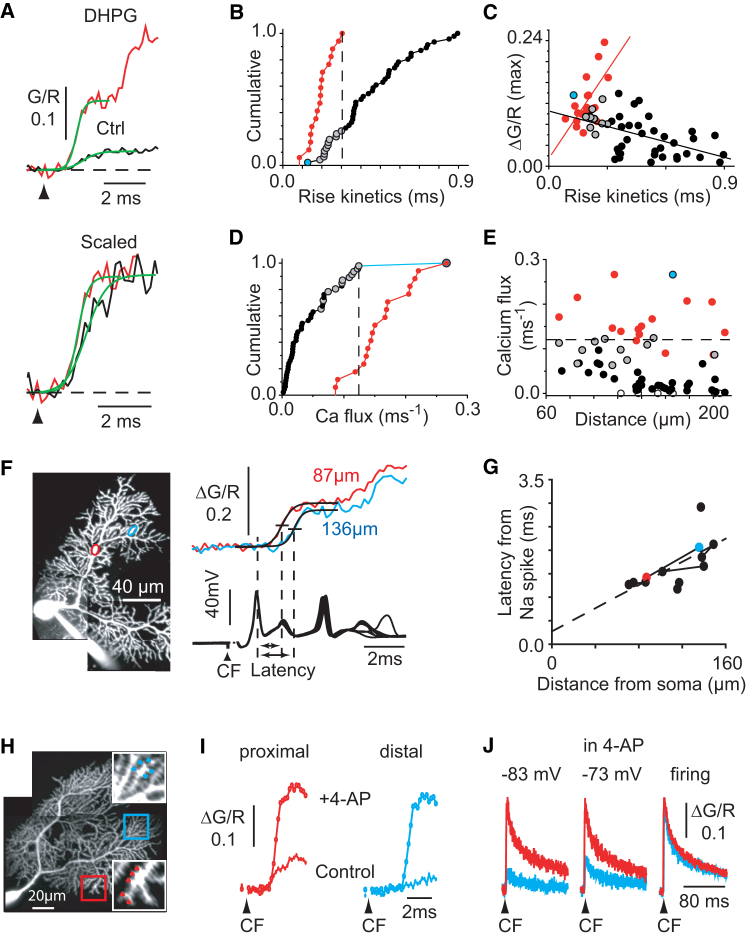
Biophysical Properties of Subthreshold and Suprathreshold Dendritic Calcium Electrogenesis (A–G) CFTCs comparison before and after 20 μM DHPG application. (A) Top: onsets of control CFCTs (500 μM Fluo-5F) (black) and first unitary transient in DHPG (red) were fitted by logistic functions (green). Bottom: scaled control CFCT and the first unitary transient, showing the faster rise of unitary transients. CF stimulation at arrowhead is shown. (B) Cumulative probability plot summarizing the rise kinetics measured with logistic fitting in (A) under control condition (n = 46; black, gray, and blue) and in the presence of DHPG (n = 17; red). Broken line indicates the slowest rise kinetics in the presence of DHPG and sets the limit between gray and black circles in control. (C) Relationship between the amplitude and the rise kinetics for the data shown in (B) with regression lines under control condition (black) and in the presence of DHPG (red). The blue point, excluded for the regression, refers to one cell in control conditions that yielded a CFCT of particularly fast rising time and a large calcium entry, probably linked to calcium spike. (D) Cumulative probability plot of calcium flux (see Results). Same color code as in (B). Broken line indicates the largest subthreshold calcium flux under control condition (0.12 ΔG/R ms^−1^). (E) Relationship between calcium flux and distance from soma. Broken line is as in (D). Six open circles correspond to very small CFCTs in control condition. (F) Simultaneous recordings of CFCTs (500 μM Fluo-5F) at a distal branchlet and a proximal dendrite (at 136 μm, blue, and 87 μm, red, from the soma, respectively), as shown in the Purkinje cell morphological reconstruction (left). Five to six POIs (9–11 successive CF stimulations) were averaged at each location. The first unitary transient evoked in 20 μM DHPG occurs at the two sites with different latencies from the CF-evoked complex spike (right). Half rise point latency (short horizontal bar) was obtained by fitting a logistic function to the rise of the unitary transient. (G) Relation between calcium spike delay and distance from the soma. Lines link paired recordings in the same cell. Broken line shows the linear regression. Colored dots correspond to data in (F). (H–J) Differential 5 μM 4-AP effect. (H) Paired recordings configuration for data shown in (I) and (J), corresponding to a distal (137 μm from soma, blue) and a proximal branchlet (108 μm from soma, red), respectively (500 μM fluo-5F; 10 POIs). (I) Superimposed traces in absence (filled circles) and presence (open circles) of 4-AP at proximal and distal sites. Cell firing rate was fixed at 30–40 Hz by hyperpolarizing current injection (control condition: averaging 20 successive CF stim, averaged simple spike frequency: 40.9 ± 4.6 Hz, averaged somatic membrane potential: −54.4 ± 0.7 mV; +4-AP condition: averaging 22 successive CF stim, 32.0 ± 4.2 Hz, −58.9 ± 0.3 mV). (J) Examples of the CFCTs recorded in the presence of 4-AP from the proximal (red, averaging eight successive CF stim) and distal (blue, averaging seven successive CF stim) dendritic sites shown in (E) at various holding membrane potentials.

The onset of control CFCTs and of the first unitary transients in DHPG (both recorded during 40 Hz spontaneous Purkinje cells firing) were fitted by a logistic function (Figure 5A), yielding an exponential steepness factor. On average, unitary transients observed in the presence of DHPG rose faster (0.19 ± 0.01 ms, exponential steepness factor of the logistic fit, n = 17) than control CFCTs (0.45 ± 0.03 ms, n = 46) (p < 0.001). However, about 25% of the control CFCTs rose as fast as unitary transients (gray circles, Figure 5B). Strikingly, the relationship between amplitude and rise kinetics (the exponential steepness factor) were opposite in control CFCTs and unitary transients (Figure 5C). The rise kinetics of control CFCTs were negatively correlated with their amplitude (slope = −0.098, r = −0.53, p < 0.0001, n = 45), as expected from the activation of T-type channels by increasingly temporally filtered electrotonic depolarizations due to cable effects. In contrast, the amplitude of unitary transients was proportional to their rise kinetics (slope = 0.56, r = 0.67, p = 0.003, n = 17), indicating that unitary transients result from regenerative events of similar peak calcium flux but variable duration.

Because the fluorescence is directly proportional to the total influx of calcium in the spine (bound calcium remains within the optical focal volume at this timescale), the derivative of the fluorescence signal is a measure of calcium flux. The peak calcium flux was calculated as the maximum slope of the CFCT, defined as the ratio of the amplitude to four times the fitted logistic exponential steepness (i.e., the derivative of the logistic function at midpoint). The peak calcium flux of unitary transients (0.16 ± 0.01 ΔG/R·ms^−1^, n = 17) (Figure 5D) was not correlated with the somatic distance (r = −0.29, p = 0.29, n = 15) (Figure 5E), confirming that dendritic calcium spikes propagate without decrement in spiny dendrites. The peak calcium flux of control CFCTs was smaller (0.04 ± 0.01 ΔG/R·ms^−1^, n = 45, p < 0.001) and its amplitude distribution only slightly overlapped with that of unitary spikes (Figure 5D). A calcium flux larger than 0.12 ΔG/R ms^−1^ can thus be considered as a hallmark of calcium spikes.

Control CFCTs with a fast rise time occurred mostly at proximal sites (gray circles, Figure 5E). The duration of calcium influx at these proximal sites (Figure 5B) is shorter than the inactivation of Cav3.1 channels (Hildebrand et al., 2009), which appear to carry most of the calcium flux (Figure 3F), and much shorter than the inactivation of P/Q channels. Hence, fast closure of T-type channels has to occur, most likely after regenerative repolarization of the proximal dendrites by a K^+^ conductance. A similar kinetic analysis cannot be performed in smooth dendrites, as intracellular diffusion of calcium will slow the fluorescence transient rise. However, the amplitude of control CFCTs (<90 μm from soma) was found to be similar to that of the first unitary spikes in DHPG (control: 0.10 ± 0.02 ΔG/R versus DHPG: 0.12 ± 0.007 ΔG/R, n = 4, p = 0.53; paired t test). These results indicate that a dampened regenerative depolarization, similar to a spikelet, may occur in the smooth dendrites and proximal spiny dendrites before mGluR1 unlocking, as observed in dendritic electrophysiological recordings (Davie et al., 2008, Kitamura and Häusser, 2011), but fails to propagate further.

### Calcium Spikes Are Generated in Proximal Dendrites Independently from Somatic Sodium Spikes

To better understand how dendritic spike unlocking can be controlled by the somatic holding potential, we determined the site of spike initiation by monitoring simultaneously the CFCTs in two spiny branchlets. In these paired optical recordings (Figure 5F), unitary transients (the first of the CFCT) always occurred earlier at proximal sites (latency from the first sodium spike 1.52 ± 0.12 ms; n = 4) than at distal sites (1.79 ± 0.19 ms, additional distance 28.2 ± 9.0 μm). This timing difference was not accounted by a change in the rise kinetics of the unitary transients (Figure 5F). When pooled from eight cells, the delay between the peak of the first sodium spike of the complex spike and the half-rise time of the first unitary transient was linearly correlated to the distance of the optical recording site from the soma with an estimated propagation speed of 81 μm ms^−1^ (r = 0.68, p = 0.016) (Figure 5G). Hence, in contrast to dendritic calcium spikes evoked by strong PF stimulations that are initiated in the stimulated distal dendrite and propagate toward the soma (Llinás et al., 1969), CF-evoked calcium spikes are initiated in proximal dendrites.

To examine whether dendritic calcium spikes were triggered directly by somatic sodium spikes within the complex spike, we determined the time of occurrence of unitary fluorescence transients in individual traces by interpolation of their half-rise point. The latencies of the first and second unitary fluorescence transients from the peak of the 1st sodium spike were 1.87 ± 0.44 ms and 4.81 ± 0.69 ms (±SD; n = 8 cells). The first unitary transient was more tightly time locked to the complex spike (jitter = SD of the latency = 379 ± 75 μs; ±SD) than the second one (jitter 550 ± 155 μs; ±SD). Cross-correlograms of the time of occurrence of somatic sodium spikes within the complex spike and of dendritic unitary calcium transients were computed (Figure S4). The correlation was not found to be significantly different (2 SD) from random correlation in four of five cells, as assessed by shuffling spikes between episodes. Hence, high-threshold calcium spikes are initiated in the proximal dendrites independently of somatic sodium spikes.

It has been proposed that the fast repolarization of spikes by Kv3 channels decreases their capacity to propagate in dendrites (Martina et al., 2003, Stuart and Häusser, 1994). We tested whether dendritic calcium spike propagation was impeded by high-threshold Kv3 potassium channels by blocking these channels with low concentrations of 4-AP (Figures S5A and S5B and Supplemental Information). The shape of the somatic complex spike was modified by 4-AP (Figures S5C–S5H), and large regenerative calcium events (Figure S5I) could be imaged in distal dendrites (Figures 5H and 5I). However, multiple spikes were never evoked (n = 10 cells; 17 branchlets) even at the most depolarized potentials, in contrast with the bursts occurring after mGluR1 activation. Furthermore, propagation of this single spike at distal sites (Figure S5J) remained regulated by the somatic membrane potential (Figures 5J and S5K). These results indicate that Kv3 channels, while involved in dendritic calcium spike repolarization, are not key in the mGluR1-mediated modulation of dendritic calcium electrogenesis.

### Identification of a Low-Threshold A-type K^+^ Conductance

**Figure 6:**
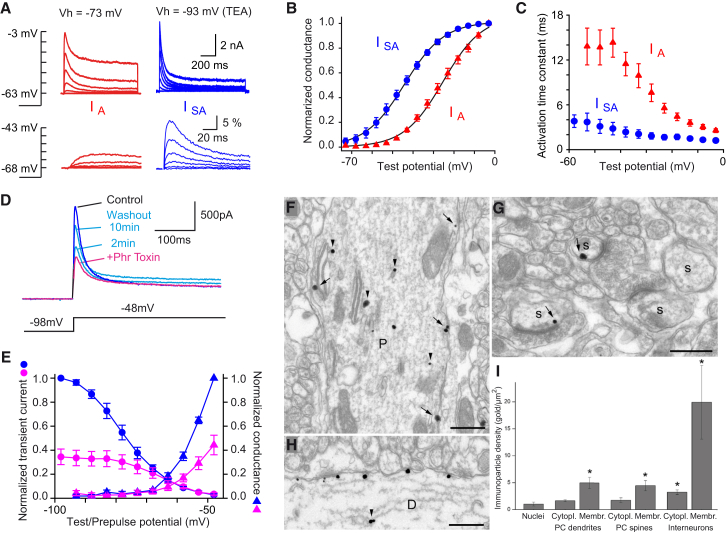
Purkinje Cells Express a Low-Threshold Inactivating K^+^ Conductance (A–C) Characteristics of the low-threshold K^+^ conductance (I_SA_) current. (A) Calcium-independent K^+^ currents evoked by 1 s test pulses to potential between −63 mV and −3 mV in 10 mV increments from a holding potential (Vh) of −73 mV (red) or −93 mV (blue). I_A_ (red) display a transient phase and/or a sustained phase slowly activated and blocked by 4 mM TEA. A low-threshold fast-activating current, fully inactivated at −73 mV (I_SA_), is recorded from Vh = −93 mV in presence of TEA (blue). Two different cells are shown. Differential rise times of the two K^+^ currents (bottom) are shown after normalization to peak amplitude at −3 mV (traces between −68 mV and −43 mV in 5 mV increments on expanded timescale). (B) Boltzmann function fit to normalized conductances of peak currents of I_A_ (seven cells, red triangles) and I_SA_ (five cells, blue circles) (see Supplemental Experimental Procedures). Note that a test potential at −50 mV will allow recording almost in isolation of I_SA_. (C) Rise time constants obtained by fitting the product of two exponential functions describing activation and inactivation to the current onset. (D and E) I_SA_ induced under physiological condition is blocked by phrixotoxin-2, a selective K_V_4 blocker. (D) Puffs of 10 μM phrixotoxin (purple trace) reversibly block I_SA_ recorded almost in isolation by step depolarization to −48 mV (see Figure 6B). (E) Normalized steady-state inactivation (circles) and activation (triangles) curves of K^+^ transient current before (blue) and after (purple) phrixotoxin application (n = 3). Inactivation was induced by changing the prepulse potential from −98 to −48 mV in 5 mV increments while keeping test potential at −48 mV, and activation curve was obtained by maintaining prepulse potential at −98 mV and changing step depolarization. Note the composite origin of traces obtained in presence of toxin: some unblocked low-threshold I_A_, plus an about 20% contaminant high-threshold I_A_ expected at – 50 mV (cf. activation curves in Figure 6B). Error bars shows ±SEM. (F and G) The plasma membrane of rat Purkinje cell dendritic shafts and spines contains immunoreactive Kv4.3 subunits. An EM micrograph shows a Purkinje cell dendrite (*P*). (F) Some gold particles (arrows) are present along the cytoplasmic side of the plasma membrane and others (arrowheads) are located in the cytoplasm. (G) Some Purkinje cell spines (*s*) are also labeled (arrows). (H) The plasma membrane of an interneuron dendrite (*D*) contains high density of gold particles. (I) Kv4.3 immunogold density values (mean ± SD, n = 4). Significant differences from nuclear background are labeled by ^∗^p < 0.05. Scale bars, 400 nm.

We looked for the molecular substrate of mGluR1 modulation and voltage-dependent spike unlocking. Because DHPG appears to regulate dendritic calcium spike initiation, it must act on voltage-gated channels activated rapidly below spike threshold. A-type potassium channels, because of their voltage-dependent inactivation, are the best candidates to modulate dendritic excitability. Two components of A-type conductances were described in Purkinje cells from young animals (Sacco and Tempia, 2002). The first can be activated from a holding potential of −73 mV by depolarizing steps above −50 mV (Figures 6A and 6B, red triangles). Addition of 4 mM TEA blocked this high-threshold A-type conductance as well as the high-threshold noninactivating Kv3 channels (Sacco and Tempia, 2002). Subsequent hyperpolarization of the holding potential from −73 mV to −93 mV revealed a second component of low-threshold A-type K^+^ conductance (I_SA_) that activated around −65 mV (Figures 6A and 6B, blue circles).

Activation of the isolated I_SA_ conductance proceeded with a *V*_*1/*2_ of −42.1 ± 0.9 mV (n = 5) and a *k* of 8.4 ± 0.2 mV (blue symbols, Figure 6B). The I_SA_ component activated in 2.8 ± 0.8 ms (n = 5) at −43 mV and in 1.2 ± 0.1 ms (n = 7) at −3 mV, much faster than the high-threshold A-type component (activation: 14.3 ± 1.9 ms at −43 mV, 2.5 ± 0.3 ms at −3 mV, n = 7) (Figures 6A, 6C, and S6A). The activation kinetics of both components was voltage dependent (exponential constant of 33.0 mV versus 23.5 mV for I_SA_ and high-threshold A-type, respectively) (Figure 6C). The inactivation of I_SA_ could be fitted by the sum of two exponential functions. The fast and slow time constants were 22.3 ± 3.4 ms (relative contribution: 69.7% ± 5.8%) (n = 5) and 96.4 ± 14.7 ms (n = 5) at −43 mV and 15.8 ± 3.6 ms (57.0% ± 3.9%) and 82.8 ± 19.1 ms (n = 5) at −3 mV (Figure S6). The time course of inactivation of the high-threshold A-type component isolated at a holding potential of −73 mV was also much slower than that of I_SA_ (116 ± 11 ms, 100%, at −43 mV and 55 ± 4 ms, 60.2% ± 4.1% at −3 mV, n = 7) (Figure S6), confirming that the two types of conductance are mediated by different channels. Hence, I_SA_ displays the properties required to implement spike gating: fast activation and large inactivation at hyperpolarized potentials.

### I_SA_ Encoded by Kv4.3 Subunits Is Expressed in Dendrites and Spines

The properties of the I_SA_ conductances are similar to those of the native and recombinant conductances encoded by the Kv4 channel family. We sought to verify that Kv4 I_SA_ conductance is the dominant K^+^ conductance activated at hyperpolarized potential under physiological conditions. Normal physiological internal and external solutions were used and K^+^ conductances were isolated by blocking Ih (10 μM ZD7288), low-threshold T-type channels (5 μM mibefradil), sodium channels (0.5 μM TTX), and GABA_A_ receptors (5 μM SR-95531). I_A_ was activated by a test potential to −48 mV, at the foot of the high threshold I_A_ activation curve (see Figure 6B), from a prepulse potential of −98 mV. These currents were reduced by 10 μM Phrixotoxin-2 (a specific blocker of Kv4 channels) applied through a local puff pipette (Figure 6E) to 44.4% ± 8.1% of control (n = 3). This block was slowly reversible in about 10 min (Figure 6D). Computing the activation and inactivation curves of the transient K^+^ current before and after toxin application (Figure 6E) confirmed that the block only affected the I_SA_ current with low inactivation threshold, while leaving untouched contaminating high-threshold I_A_ most noticeable at prepulse potentials of −63 mV and above (about 20% of total control current).

Kv4.3 mRNA expression has been reported in Purkinje cells (Serôdio et al., 1996). The protein is abundantly expressed in the molecular layer (Amarillo et al., 2008) and is found at high levels at specialized junctions made between CFs and molecular layer interneurons (Kollo et al., 2006). Pre-embedding immunogold reactions were carried out to investigate whether the Kv4.3 subunit of A-type potassium channels is also present on the plasma membrane of rat Purkinje cells. Gold particle densities along the plasma membrane of Purkinje cell dendritic shafts and spines were significantly (p < 0.001) higher than the nonspecific background labeling measured over the nuclei, indicating that the plasma membranes of Purkinje cells contain the Kv4.3 subunit (Figures 6F and 6G). This quantitative analysis also confirmed the significant labeling of interneuron plasma membranes, as shown previously (Kollo et al., 2006) (Figure 6H). No significant difference between the labeling intensity of Purkinje cell dendritic shafts and spines was found (Figure 6I). The presence of Kv4.3 subunits in Purkinje cell spine and dendritic shaft plasma membranes was also demonstrated in P22 mouse with SDS-digested freeze-fracture replica-immunolabeling technique in cerebellum (Figure S7).

### DHPG Causes a Hyperpolarizing Shift of the Inactivation of Kv4 Channels

**Figure 7:**
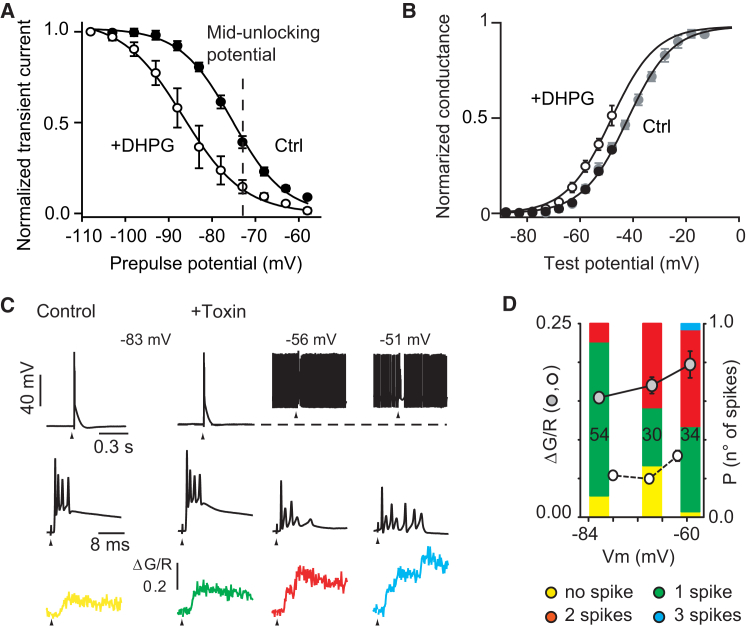
K_V_4 Channels Downregulation Underlies Spike Unlocking (A) Steady-state inactivation curves of I_SA_ currents recorded under physiological condition in the absence (filled circle, n = 8) and presence (open circle, n = 4) of 20 μM DHPG. For the inactivation curves, the amplitude of the transient current evoked by steps to −48 mV after 1 s prepulses to potential from −108 to −58 mV in 5 mV increments were normalized to the amplitude obtained by prepulse at −108 mV. These results were plotted against the prepulse potential and fitted using a Boltzmann function (see Results). (B) Normalized conductances of the transient currents evoked from Vh = −98 mV in the absence (black circle, n = 8) and the presence (open circle, n = 4) of 20 μM DHPG. Boltzmann fits are shown by continuous line. For control currents, fit is according to Figure 6B data (gray circle, n = 5). For data in presence of DHPG, see Results. Error bars show ±SEM. (C) Phrixotoxin application induces spike-like CFCTs. Examples of the electrophysiological traces (top, and middle on expanded timescale) and corresponding CFCTs (bottom traces) when applying a CF stimulation (at arrowhead) in the absence (yellow) and the presence (green, red, and blue) of 2 μM toxin (single stimulations). Color code of calcium spikes number is as in (D). Broken line indicates −83 mV. (D) Averaged data from three cells recorded in absence and presence of 1–2 μM phrixotoxin (white and gray circles, respectively). Note the only slight sensitivity of the discharge to membrane potential (compared to the DHPG-induced sensitivity in Figure 3D).

Using the same near-physiological isolation conditions as in Figures 6A–6E, we tested whether mGluR1 activation modulates Kv4 conductance. Application of DHPG shifted the midinactivation of the Kv4 channels from −75.3 ± 0.7 mV to −86.3 ± 2.3 mV (p = 0.008) without changing the inactivation slope (from −5.9 ± 0.4 mV to −5.9 ± 0.5 mV, p = 0.933) (Figure 7A). The activation curve (Figure 7B) was also shifted by 6 mV toward a hyperpolarized potential (as deduced by fitting Boltzmann equations to the partial activation curves and normalizing to the extrapolated maximal transient current deduced from the I_SA_ data in Figure 6B). The leftward shift in the inactivation curve will decrease the available Kv4 conductance at all holding potentials ranging from −100 mV to −60 mV. At midunlocking potential for the calcium spikes (−72 mV; see Figure 3F) the available conductance is reduced by more than 60%. In conclusion, the shift of 11 mV in the Kv4 inactivation curve appears large enough to explain the voltage-dependent spike unlocking induced by DHPG (Figures 3F).

**Figure 8:**
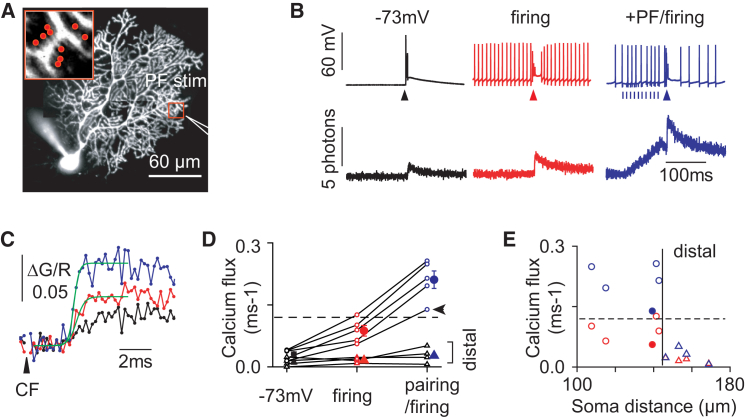
Local Initiation of Spike-like CFCTs in the Purkinje Cell Dendrite by Concomitant PF and CF Stimulations (A) Optical recording sites (139 μm from soma) and glass pipette position for PF stimulation, for data in (B) and (C). Inset shows the nine POIs, all placed on spines activated by PF stimulation. (B) Somatic membrane potential (top) and averaged CFCTs (bottom) under three conditions: single CF stimulus (arrowhead) at Vh = −73 mV (black traces), under spontaneous firing (0 pA holding current) (red traces), and 20 ms after ten PF stimuli (vertical short line) at 100 Hz under spontaneous firing (0 pA holding current) (blue traces). CFCTs represent averages over 10, 5, and 9 CF stimuli, respectively. Note that the CFCT is increased by depolarization (Figures 3E and 3F) and further transformed into a spike-like CFCT by a preceding PF stimulation. (C) Superimposed CFTCs recorded in (B) to show at expanded timescale the distinct onset kinetics estimated by fitting logistic functions (green). (D) Calcium flux evaluated from nine spiny branchlets in eight cells. Colors indicate the recording conditions, as in (B). Data from the same spiny branchlet are connected with lines: open circles and open triangles (spiny branchlets selected for their terminal distal position). Arrowhead points to recordings illustrated in (A)–(C) and to filled circles in (E). Filled circles and triangles represent averaged data under each condition. Broken line indicates the criteria for calcium spike, obtained in Figure 5D. Error bars represent SEM. (E) Same data as in (D) with (blue) or without (red) pairing of PF stimuli preceding the CF stimulation as a function of the distance from soma. Vertical line indicates the largest distance to soma from which a calcium spike was evoked (∼140 μm).

If Kv4 inactivation underlies the voltage and mGluR1 dependence of spike unlocking, blocking Kv4 conductance with Phrixotoxin should produce constitutive voltage-independent spike unlocking. Application of 1–2 μM toxin through a local superfusion pipette led to a strong potentiation of the CFCT (0.047 ± 0.004 ΔG/R at −77 ± 0.4 mV in control, n = 103 CF stimulations; 0.155 ± 0.006 ΔG/R at −79 ± 0.6 mV, n = 44 CF stimulations; p < 0.001, n = 3 cells) and to the appearance of high-threshold spike bursts (one to three spikes) in the distal dendrites of three out of five cells (Figure 7C). The other cells only displayed a mild increase in the calcium transient amplitude, probably due to insufficient penetration of the toxin in the slice. As anticipated, the voltage-dependence of the spike number and of the peak CFCT amplitude in the three cells responding to the toxin was greatly reduced compared to DHPG (Figures 8D and 3F) (22% decrease of CFCT amplitude and 36% decrease in number of spike between −59 ± 0.5 mV and −81 ± 0.5 mV in Phrixotoxin; 80% decrease of CFCT amplitude and 94% decrease in number of spike between −62 ± 0.1 mV and −80 ± 0.3 mV in DHPG). Hence, activation of low-threshold Kv4 channels limits the initiation of high-threshold spike in proximal dendrites during CF-evoked dendritic EPSPs. Increased inactivation of Kv4 channels by activity-dependent signals (depolarization and mGluR1 activation) can fully account for the observed dendritic spike unlocking.

### PF Beam Stimulation of Spiny Dendrites Unlocks CF-Evoked Calcium Spikes

Because PF stimulations can both activate mGluR1 receptors and depolarize the distal dendrites, we tested whether PF stimulations similar to the ones used for LTD induction protocols could produce spike unlocking. As previously shown (Hildebrand et al., 2009), a burst of ten PF beam stimulations at 100–200 Hz produced a calcium transient mediated by T-type voltage-gated calcium channels in a circumscribed region of the dendrites. Pairing of the PF stimulation with the CF stimulation (5–20 ms after PF offset) at depolarized potentials (spontaneous firing) increased the CFCT measured within the PF responsive region from 0.071 ± 0.006 ΔG/R to 0.094 ± 0.005 ΔG/R (p = 0.016, paired t test, n = 5) (Figures 8B and 8C). This potentiation resulted in a calcium flux shift from subthreshold regime (0.087 ± 0.013 ΔG/R·ms^−1^) to suprathreshold regime (0.211 ± 0.021 ΔG/R·ms^−1^) in all the cells (n = 5) (Figures 8C and 8D, circles). Multiple spikes were never observed. This milder effect can be explained by the persistence of Kv4 channels in the dendrites outside of the stimulated PF beam.

The spatial restriction of the effect was further tested by PF stimulation of extremely distal spiny branchlets (soma distance above 150 μm). At these locations, the sensitivity to somatic depolarization appeared reduced (hyperpolarized 0.021 ± 0.008 ΔG/R, depolarized 0.031 ± 0.005 ΔG/R) and the CFCT was only mildly potentiated by PF pairing (0.039 ± 0.004 ΔG/R n = 4), remaining well below spike threshold (control calcium flux 0.017 ± 0.003 ΔG/R·ms^−1^; paired calcium flux 0.028 ± 0.009 ΔG/R·ms^−1^) (Figures 8D and 8E, triangles). Hence, focal PF beam stimulations can unlock local nonpropagated CF induced P/Q spikes but only if the PF input is not too remote from the proximal initiation sites in the smooth dendrites. Widespread PF input over the whole dendritic tree would probably be necessary to achieve global unlocking.

## Discussion

We used RAMP microscopy to map CFCTs at high temporal resolution and resolve calcium spikes in optical recordings from Purkinje cell spiny dendrites. In contrast to the stereotypical somatic complex spike, we find that dendritic calcium electrogenesis is a regulated process. In a subthreshold regime, calcium influx decreases with distance from the soma and is mediated by T-type channels activation. In a suprathreshold regime, bursts of P/Q calcium spikes propagate from the smooth dendrites to the spiny branchlets. The gating between these two regimes is under the control of two activity-dependent signals, mGluR1 activation and Purkinje cell depolarization. Kv4.3 channel modulation by mGluR1 mediates this gating.

### Optical Recording of Calcium Electrogenesis in Purkinje Cell Dendrites

Whether small-amplitude short-lasting spikelets in Purkinje cell smooth dendrites (Davie et al., 2008, Fujita, 1968, Kitamura and Häusser, 2011, Llinás and Hess, 1976, Rancz and Häusser, 2006) are caused by actual regenerative propagated calcium spikes has remained unclear. Our optical recordings suggest that fast-repolarizing events may occur in smooth dendrites and proximal spiny dendrites in basal conditions but fail to propagate distally as full-blown spikes. The associated CFCT decreases with distance from the soma, reaching undetectable levels in distal dendrites, as previously suggested by wide-field imaging data (Miyakawa et al., 1992, Ross and Werman, 1987). Spikelets may thus represent failed regenerative events crowning the large CF excitatory postsynaptic current (EPSC). Interestingly, previous dendritic recordings indicate that CF stimulations evoke a single spikelet, only rarely followed by a second one (Davie et al., 2008, Kitamura and Häusser, 2011, Llinás and Sugimori, 1980), as expected for local regenerative amplification at the peak of the CF EPSC. Strong PF stimulations can also produce local calcium influx mediated by high-threshold P/Q channels (Rancz and Häusser, 2006), which are recorded as spikelets from the nearby smooth dendrites (Rancz and Häusser, 2006), further supporting that low-amplitude spikelets recorded electrophysiologically cannot be unambiguously associated with the occurrence of high-threshold propagated dendritic calcium spikes.

Electrophysiological techniques fail to provide accurate measure of the time course of fast regenerative events in dendrites, due to filtering and dampening by leak, pipette access resistance, and capacitive load. The temporal resolution of optical recordings of calcium transients is defined by the time constant of calcium binding to the dye, which is approximately 2 μs for 500 μM Fluo5F, assuming a k_on_ of 10^9^ M^−1^ s^−1^ (Lattanzio and Bartschat, 1991). The stimulus-evoked change in fluorescence is linearly related to the cumulative Ca influx up to the dye concentration (Higley and Sabatini, 2008). Using these advantages, we provide unambiguous description of nondecremental, all-or-none, high-threshold calcium spikes mediated by P/Q type channels. The calculated charge corresponding to a calcium spike is 3.6 fC entering each spine, with a half-time of 400 μs (see Supplemental Information). This would depolarize the spine by 180 mV, strongly suggesting that calcium spikes are overshooting in spiny branchlets, unlike spikelets recorded electrophysiologically (Davie et al., 2008, Fujita, 1968, Rancz and Häusser, 2006).

High-rate paired optical recordings indicate that CFCTs propagate at a speed of 80 μm.ms^−1^, slightly slower than assessed from field potential recordings in vivo (Llinás and Hess, 1976, Llinás et al., 1968). After full unlocking of the dendrites by mGluR1 activation and depolarization, CFCTs are composed of high-frequency bursts (500 Hz) of calcium spikes, consistent with graded variations of global CFCT amplitudes previously reported at lower temporal resolution (Miyakawa et al., 1992, Ross and Werman, 1987). Variability of the number of spikes in each burst or failure of spikes to propagate in some dendritic branches may arise from the stochastic nature of P/Q channels activation (Anwar et al., 2013).

### Mechanisms Underlying Dendritic Spike Gating

In pyramidal neurons, fast activation of a low-threshold A-type K^+^ conductance (I_SA_) controls the capacity of spikes to back propagate in distal dendrites (Hoffman et al., 1997). In Purkinje cells, the potentiating effect of strong somatic depolarizations (Cavelier et al., 2002, Chan et al., 1989) and that of direct field depolarization (Midtgaard et al., 1993) on calcium transients and spikes evoked by CF and PF stimulation has also been tentatively attributed to the inactivation of an unidentified dendritic A-type or delayed conductance. Dendrotoxin-sensitive, Kv1-encoded, dendritic A-type conductances have been shown to modulate somatic sodium spike rate and control the duration of the complex spike (Khavandgar et al., 2005, McKay et al., 2005) in Purkinje cells. Our data rule out the role of these channels in gating dendritic spikes. We show that the Kv4.3 subunit is present in Purkinje cell spines and shafts and mediate a fast-activating I_SA_. The block of this I_SA_ by phrixotoxin unlocks dendritic calcium spikes, as mGluR1 activation does. By shifting the inactivation curve of I_SA_ toward hyperpolarized potentials, mGluR1 activation decreases the availability of these channels at Purkinje cell resting membrane potential and favors both the proximal initiation of calcium spikes and their propagation into spiny dendrites. Membrane potential may then influence calcium spike genesis in two distinct ways. First the somatic membrane potential imposes a bias on the spike initiation site, thus controlling the number of calcium spikes emitted on top of the CF EPSP. Second, somatic depolarization preceding the CF EPSP can spread electrotonically (Roth and Häusser, 2001) and increase the inactivation of Kv4.3 channels in spiny dendrites, favoring calcium spike initiation and propagation. Direct synaptic control of dendritic membrane potential by inhibitory interneurons has been shown to inhibit CF calcium signaling (Callaway et al., 1995, Kitamura and Häusser, 2011). We propose that the effect of synaptic hyperpolarization may be amplified by an increase in I_SA_ availability through recovery from inactivation.

### Molecular Layer Activity and Activation of mGluR1 Receptors

mGluR1 receptors are activated at PF synapses by high-frequency granule cell firing (Finch and Augustine, 1998, Marcaggi et al., 2009, Takechi et al., 1998), similar to those produced in vivo by physiological patterns of activity (Barmack and Yakhnitsa, 2008, Bengtsson and Jörntell, 2009, Chadderton et al., 2004, Ekerot and Jörntell, 2008, Rancz et al., 2007). Given the long time course of metabotropic effects, physiological levels of granule cell activity may maintain a substantial level of mGluR1 signaling (Marcaggi et al., 2009), crosstalk between GABAB, and mGluR1 receptors activation (Hirono et al., 2001) adding integration of molecular layer interneurons activity. Pooling of glutamate between multiple CFs by spillover (Szapiro and Barbour, 2007) may also “contribute” to widespread mGluR1 tone in the molecular layer during local CF synchrony (Ozden et al., 2009). It is therefore likely that spike unlocking by mGluR1 occurs at physiological levels of molecular layer activity.

CFCTs have been recorded in the distal dendrites of Purkinje cells in vivo (Ozden et al., 2009, Schultz et al., 2009, Sullivan et al., 2005). However, in the absence of pharmacological data or high-frequency optical recordings, it remains unclear whether these CFCTs arise from subthreshold T-type channels activation or from propagated P/Q spikes. Quantitative measurements of the CFCTs have been obtained in the anesthetized animal during membrane voltage manipulations (Kitamura and Häusser, 2011). In that study, CFCT potentiation by depolarization is modest, except for extreme depolarized plateau potentials, and therefore similar to the voltage dependence that we report in absence of DHPG. This is consistent with granule cell activity being reduced in the anesthetized animal (Bengtsson and Jörntell, 2007). Elevated PF activity found in the behaving animal is probably necessary to unlock dendritic calcium spikes.

### CF Graded Calcium Signaling and Cerebellar Learning

Strong high-frequency PF beam stimulations can produce local (Canepari and Vogt, 2008, Rancz and Häusser, 2006) or propagated (Llinás et al., 1969) calcium spikes. However, milder stimulations at similar frequencies will only produce a smaller, T-mediated, local calcium influx (Brenowitz and Regehr, 2005, Wang et al., 2000) that can be restricted to individual spines (Denk et al., 1995, Hildebrand et al., 2009). T-type signaling is required for the induction of long-term potentiation at PF synapses by trains of PF stimulations (Ly et al., 2013). Pairing mild PF stimulations with CF stimulations will evoke local dendritic calcium transients that are much larger than those triggered by CF stimulations alone (Brenowitz and Regehr, 2005, Canepari and Vogt, 2008, Wang et al., 2000) and that have been used to trigger short-term (Brenowitz and Regehr, 2005) and long-term (Canepari and Vogt, 2008, Ito and Kano, 1982, Wang et al., 2000) plasticity.

The mechanisms underlying associative CF/PF calcium signaling are not well understood. High-frequency PF bursts activate postsynaptic mGluR1s (Finch and Augustine, 1998, Takechi et al., 1998). The subsequent mobilization of IP3-sensitive calcium stores by the CF-mediated calcium transient (Sarkisov and Wang, 2008), as a result of the calcium dependence of IP3 receptors, has been proposed to mediate associative calcium signaling and plasticity (Miyata et al., 2000, Wang et al., 2000). However, supralinear summation of calcium transients during associative PF-CF stimulations is also regulated by the membrane potential (Brenowitz and Regehr, 2005, Canepari and Vogt, 2008) and becomes mGluR1-independent for larger PF stimulations, suggesting the involvement of voltage-gated processes (Wang et al., 2000) upstream of store release. Furthermore, IP3 stores are not required for the induction of short-term depression by associative PF-CF stimulations (Brenowitz and Regehr, 2005). We show here that PF-CF paired stimulations may unlock calcium spikes locally in Purkinje cell dendrites through voltage-dependent Kv4 channel modulation. However, global molecular layer activity in addition to local stimulation is probably required to achieve widespread dendritic unlocking and dendritic spike propagation.

Our findings suggest a framework for activity-dependent cerebellar learning. First, increased activity in the molecular layer will favor calcium spikes and PF synaptic depression, playing a homeostatic role. Second, Purkinje cell discharge rate will gate calcium spikes and thus synaptic plasticity. Transitions to a hyperpolarized state (Loewenstein et al., 2005, Williams et al., 2002) may prevent the induction of synaptic plasticity, for example, in Purkinje cells that are not used by ongoing motor tasks. Decreased PF synapse depression at reduced firing rate may prevent learning saturation. Furthermore, our results suggest a mechanism by which synaptic plasticity may be induced by altered PF or Purkinje cell activity, even with unaltered CF activity, as recently shown during vestibulo-ocular learning protocols (Ke et al., 2009). Finally, gating calcium spikes offers a substrate for metaplasticity, as in hippocampal neurons (Losonczy et al., 2008), through long-term regulations of Purkinje cell dendritic excitability, as observed following learning protocols in vivo (Schreurs et al., 1998) and in vitro (Belmeguenai et al., 2010).

## Experimental Procedures

### Calcium Imaging

CFCTs were monitored at high speed (kHz) by two-photon random-access microscopy, using acousto-optic deflector (AOD)-based scanning (Otsu et al., 2008). Two-photon excitation was produced by an infrared Ti-Sa pulsed laser (Tsunami pumped by a 6 W Millenia VI, 400 mW output at 700 fs, Spectra-Physics) tuned to 825 nm. A custom-made user interface programmed under Labview was used to coordinate scanning protocols and signal acquisition. Fluorescence photons were detected by a cooled AsGaP photomultiplier (H7421-40, Hamamatsu) discriminated and counted on a fast digital card. Externally triggered episodes of 500–1,500 points (100 ms–1.3 s) were used to avoid phototoxicity.

Relative fluorescence was expressed as ΔG/R, i.e., variations in Fluo-4 or Fluo-5F signals change (ΔG) divided by calcium-independent Alexa 594 fluorescence (R). This ratiometric method scales the calcium fluorescence signal to the volume of the imaged compartment yielding a measurement of the dye-bound cytoplasmic calcium concentration independent of the dendritic geometry. To monitor basal Ca^2+^, we used Go/R, where Go is the basal fluorescence before CF stimulation.

Experiments were carried in compliance with the ethic recommendations of the CNRS.

For additional information, see online Supplemental Experimental Procedures.

## Authors Contributions

S.D. and B.M. built the RAMP microscope. Y.O., P.M., P.I., A.F., and S.D. designed the study and performed the imaging and electrophysiology experiments and the analysis. M. Kollo and Z.N. performed the immunolocalization experiments. M. Kano, M.T., and K.S. generated the Cav3.1 KO mice. Y.O., P.M., P.I., A.F., and S.D. wrote the paper.

## Supplemental Information

Document S1. Supplemental Experimental Procedure and Figures S1–S7

Document S2. Article plus Supplemental Information

## References

[bib1] Amarillo Y., De Santiago-Castillo J.A., Dougherty K., Maffie J., Kwon E., Covarrubias M., Rudy B. (2008). Ternary Kv4.2 channels recapitulate voltage-dependent inactivation kinetics of A-type K+ channels in cerebellar granule neurons. J. Physiol..

[bib2] Anwar H., Hepburn I., Nedelescu H., Chen W., De Schutter E. (2013). Stochastic calcium mechanisms cause dendritic calcium spike variability. J. Neurosci..

[bib3] Barmack N.H., Yakhnitsa V. (2008). Functions of interneurons in mouse cerebellum. J. Neurosci..

[bib4] Belmeguenai A., Hosy E., Bengtsson F., Pedroarena C.M., Piochon C., Teuling E., He Q., Ohtsuki G., De Jeu M.T., Elgersma Y. (2010). Intrinsic plasticity complements long-term potentiation in parallel fiber input gain control in cerebellar Purkinje cells. J. Neurosci..

[bib5] Bengtsson F., Jörntell H. (2007). Ketamine and xylazine depress sensory-evoked parallel fiber and climbing fiber responses. J. Neurophysiol..

[bib6] Bengtsson F., Jörntell H. (2009). Sensory transmission in cerebellar granule cells relies on similarly coded mossy fiber inputs. Proc. Natl. Acad. Sci. USA.

[bib7] Brenowitz S.D., Regehr W.G. (2005). Associative short-term synaptic plasticity mediated by endocannabinoids. Neuron.

[bib8] Callaway J.C., Lasser-Ross N., Ross W.N. (1995). IPSPs strongly inhibit climbing fiber-activated [Ca2+]i increases in the dendrites of cerebellar Purkinje neurons. J. Neurosci..

[bib9] Canepari M., Vogt K.E. (2008). Dendritic spike saturation of endogenous calcium buffer and induction of postsynaptic cerebellar LTP. PLoS ONE.

[bib10] Cavelier P., Pouille F., Desplantez T., Beekenkamp H., Bossu J.L. (2002). Control of the propagation of dendritic low-threshold Ca(2+) spikes in Purkinje cells from rat cerebellar slice cultures. J. Physiol..

[bib11] Chadderton P., Margrie T.W., Häusser M. (2004). Integration of quanta in cerebellar granule cells during sensory processing. Nature.

[bib12] Chan C.Y., Hounsgaard J., Midtgaard J. (1989). Excitatory synaptic responses in turtle cerebellar Purkinje cells. J. Physiol..

[bib13] Cornelisse L.N., van Elburg R.A., Meredith R.M., Yuste R., Mansvelder H.D. (2007). High speed two-photon imaging of calcium dynamics in dendritic spines: consequences for spine calcium kinetics and buffer capacity. PLoS ONE.

[bib14] Davie J.T., Clark B.A., Häusser M. (2008). The origin of the complex spike in cerebellar Purkinje cells. J. Neurosci..

[bib15] Denk W., Sugimori M., Llinás R. (1995). Two types of calcium response limited to single spines in cerebellar Purkinje cells. Proc. Natl. Acad. Sci. USA.

[bib16] Ekerot C.F., Jörntell H. (2008). Synaptic integration in cerebellar granule cells. Cerebellum.

[bib17] Etzion Y., Grossman Y. (1998). Potassium currents modulation of calcium spike firing in dendrites of cerebellar Purkinje cells. Exp. Brain Res..

[bib18] Finch E.A., Augustine G.J. (1998). Local calcium signalling by inositol-1,4,5-trisphosphate in Purkinje cell dendrites. Nature.

[bib19] Fujita Y. (1968). Activity of dendrites of single Purkinje cells and its relationship to so-called inactivation response in rabbit cerebellum. J. Neurophysiol..

[bib20] Higley M.J., Sabatini B.L. (2008). Calcium signaling in dendrites and spines: practical and functional considerations. Neuron.

[bib21] Hildebrand M.E., Isope P., Miyazaki T., Nakaya T., Garcia E., Feltz A., Schneider T., Hescheler J., Kano M., Sakimura K. (2009). Functional coupling between mGluR1 and Cav3.1 T-type calcium channels contributes to parallel fiber-induced fast calcium signaling within Purkinje cell dendritic spines. J. Neurosci..

[bib22] Hirono M., Yoshioka T., Konishi S. (2001). GABA(B) receptor activation enhances mGluR-mediated responses at cerebellar excitatory synapses. Nat. Neurosci..

[bib23] Hoffman D.A., Magee J.C., Colbert C.M., Johnston D. (1997). K+ channel regulation of signal propagation in dendrites of hippocampal pyramidal neurons. Nature.

[bib24] Ito M., Kano M. (1982). Long-lasting depression of parallel fiber-Purkinje cell transmission induced by conjunctive stimulation of parallel fibers and climbing fibers in the cerebellar cortex. Neurosci. Lett..

[bib25] Ke M.C., Guo C.C., Raymond J.L. (2009). Elimination of climbing fiber instructive signals during motor learning. Nat. Neurosci..

[bib26] Khavandgar S., Walter J.T., Sageser K., Khodakhah K. (2005). Kv1 channels selectively prevent dendritic hyperexcitability in rat Purkinje cells. J. Physiol..

[bib27] Kitamura K., Häusser M. (2011). Dendritic calcium signaling triggered by spontaneous and sensory-evoked climbing fiber input to cerebellar Purkinje cells in vivo. J. Neurosci..

[bib28] Kollo M., Holderith N.B., Nusser Z. (2006). Novel subcellular distribution pattern of A-type K+ channels on neuronal surface. J. Neurosci..

[bib29] Larkum M.E., Zhu J.J., Sakmann B. (1999). A new cellular mechanism for coupling inputs arriving at different cortical layers. Nature.

[bib30] Lattanzio F.A., Bartschat D.K. (1991). The effect of pH on rate constants, ion selectivity and thermodynamic properties of fluorescent calcium and magnesium indicators. Biochem. Biophys. Res. Commun..

[bib31] Llinás R., Hess R. (1976). Tetrodotoxin-resistant dendritic spikes in avian Purkinje cells. Proc. Natl. Acad. Sci. USA.

[bib32] Llinás R., Sugimori M. (1980). Electrophysiological properties of in vitro Purkinje cell dendrites in mammalian cerebellar slices. J. Physiol..

[bib33] Llinás R., Nicholson C., Freeman J.A., Hillman D.E. (1968). Dendritic spikes and their inhibition in alligator Purkinje cells. Science.

[bib34] Llinás R., Nicholson C., Precht W. (1969). Preferred centripetal conduction of dendritic spikes in alligator Purkinje cells. Science.

[bib35] Loewenstein Y., Mahon S., Chadderton P., Kitamura K., Sompolinsky H., Yarom Y., Häusser M. (2005). Bistability of cerebellar Purkinje cells modulated by sensory stimulation. Nat. Neurosci..

[bib36] Losonczy A., Makara J.K., Magee J.C. (2008). Compartmentalized dendritic plasticity and input feature storage in neurons. Nature.

[bib37] Ly R., Bouvier G., Schonewille M., Arabo A., Rondi-Reig L., Léna C., Casado M., De Zeeuw C.I., Feltz A. (2013). T-type channel blockade impairs long-term potentiation at the parallel fiber-Purkinje cell synapse and cerebellar learning. Proc. Natl. Acad. Sci. USA.

[bib38] Maejima T., Oka S., Hashimotodani Y., Ohno-Shosaku T., Aiba A., Wu D., Waku K., Sugiura T., Kano M. (2005). Synaptically driven endocannabinoid release requires Ca2+-assisted metabotropic glutamate receptor subtype 1 to phospholipase Cbeta4 signaling cascade in the cerebellum. J. Neurosci..

[bib39] Magee J.C., Johnston D. (2005). Plasticity of dendritic function. Curr. Opin. Neurobiol..

[bib40] Marcaggi P., Mutoh H., Dimitrov D., Beato M., Knöpfel T. (2009). Optical measurement of mGluR1 conformational changes reveals fast activation, slow deactivation, and sensitization. Proc. Natl. Acad. Sci. USA.

[bib41] Martina M., Yao G.L., Bean B.P. (2003). Properties and functional role of voltage-dependent potassium channels in dendrites of rat cerebellar Purkinje neurons. J. Neurosci..

[bib42] McDonough S.I., Bean B.P. (1998). Mibefradil inhibition of T-type calcium channels in cerebellar purkinje neurons. Mol. Pharmacol..

[bib43] McKay B.E., Turner R.W. (2004). Kv3 K+ channels enable burst output in rat cerebellar Purkinje cells. Eur. J. Neurosci..

[bib44] McKay B.E., Molineux M.L., Mehaffey W.H., Turner R.W. (2005). Kv1 K+ channels control Purkinje cell output to facilitate postsynaptic rebound discharge in deep cerebellar neurons. J. Neurosci..

[bib45] Midtgaard J., Lasser-Ross N., Ross W.N. (1993). Spatial distribution of Ca2+ influx in turtle Purkinje cell dendrites in vitro: role of a transient outward current. J. Neurophysiol..

[bib46] Miyakawa H., Lev-Ram V., Lasser-Ross N., Ross W.N. (1992). Calcium transients evoked by climbing fiber and parallel fiber synaptic inputs in guinea pig cerebellar Purkinje neurons. J. Neurophysiol..

[bib47] Miyata M., Finch E.A., Khiroug L., Hashimoto K., Hayasaka S., Oda S.I., Inouye M., Takagishi Y., Augustine G.J., Kano M. (2000). Local calcium release in dendritic spines required for long-term synaptic depression. Neuron.

[bib48] Otsu Y., Bormuth V., Wong J., Mathieu B., Dugué G.P., Feltz A., Dieudonné S. (2008). Optical monitoring of neuronal activity at high frame rate with a digital random-access multiphoton (RAMP) microscope. J. Neurosci. Methods.

[bib49] Ozden I., Sullivan M.R., Lee H.M., Wang S.S. (2009). Reliable coding emerges from coactivation of climbing fibers in microbands of cerebellar Purkinje neurons. J. Neurosci..

[bib50] Rancz E.A., Häusser M. (2006). Dendritic calcium spikes are tunable triggers of cannabinoid release and short-term synaptic plasticity in cerebellar Purkinje neurons. J. Neurosci..

[bib51] Rancz E.A., Ishikawa T., Duguid I., Chadderton P., Mahon S., Häusser M. (2007). High-fidelity transmission of sensory information by single cerebellar mossy fibre boutons. Nature.

[bib52] Ross W.N., Werman R. (1987). Mapping calcium transients in the dendrites of Purkinje cells from the guinea-pig cerebellum in vitro. J. Physiol..

[bib53] Roth A., Häusser M. (2001). Compartmental models of rat cerebellar Purkinje cells based on simultaneous somatic and dendritic patch-clamp recordings. J. Physiol..

[bib54] Sacco T., Tempia F. (2002). A-type potassium currents active at subthreshold potentials in mouse cerebellar Purkinje cells. J. Physiol..

[bib55] Sarkisov D.V., Wang S.S. (2008). Order-dependent coincidence detection in cerebellar Purkinje neurons at the inositol trisphosphate receptor. J. Neurosci..

[bib56] Schreurs B.G., Gusev P.A., Tomsic D., Alkon D.L., Shi T. (1998). Intracellular correlates of acquisition and long-term memory of classical conditioning in Purkinje cell dendrites in slices of rabbit cerebellar lobule HVI. J. Neurosci..

[bib57] Schultz S.R., Kitamura K., Post-Uiterweer A., Krupic J., Häusser M. (2009). Spatial pattern coding of sensory information by climbing fiber-evoked calcium signals in networks of neighboring cerebellar Purkinje cells. J. Neurosci..

[bib58] Serôdio P., Vega-Saenz de Miera E., Rudy B. (1996). Cloning of a novel component of A-type K+ channels operating at subthreshold potentials with unique expression in heart and brain. J. Neurophysiol..

[bib59] Sjöström P.J., Rancz E.A., Roth A., Häusser M. (2008). Dendritic excitability and synaptic plasticity. Physiol. Rev..

[bib60] Stuart G., Häusser M. (1994). Initiation and spread of sodium action potentials in cerebellar Purkinje cells. Neuron.

[bib61] Sullivan M.R., Nimmerjahn A., Sarkisov D.V., Helmchen F., Wang S.S. (2005). In vivo calcium imaging of circuit activity in cerebellar cortex. J. Neurophysiol..

[bib62] Szapiro G., Barbour B. (2007). Multiple climbing fibers signal to molecular layer interneurons exclusively via glutamate spillover. Nat. Neurosci..

[bib63] Takechi H., Eilers J., Konnerth A. (1998). A new class of synaptic response involving calcium release in dendritic spines. Nature.

[bib64] Tank D.W., Sugimori M., Connor J.A., Llinás R.R. (1988). Spatially resolved calcium dynamics of mammalian Purkinje cells in cerebellar slice. Science.

[bib65] Usowicz M.M., Sugimori M., Cherksey B., Llinás R. (1992). P-type calcium channels in the somata and dendrites of adult cerebellar Purkinje cells. Neuron.

[bib66] Wang S.S., Denk W., Häusser M. (2000). Coincidence detection in single dendritic spines mediated by calcium release. Nat. Neurosci..

[bib67] Williams S.R., Christensen S.R., Stuart G.J., Häusser M. (2002). Membrane potential bistability is controlled by the hyperpolarization-activated current I(H) in rat cerebellar Purkinje neurons in vitro. J. Physiol..

[bib68] Womack M.D., Khodakhah K. (2004). Dendritic control of spontaneous bursting in cerebellar Purkinje cells. J. Neurosci..

