# Supplementary material for: Activity-Dependent Gating of Calcium Spikes by A-type K+ Channels Controls Climbing Fiber Signaling in Purkinje Cell Dendrites
Source: Neuron. 2014 Oct 1;84(1):137–51. doi: 10.1016/j.neuron.2014.08.035 (PMC4183427; doi:10.1016/j.neuron.2014.08.035)
Supplement: Document S1. Supplemental Experimental Procedure and Figures S1–S7 [file mmc1.pdf]

Neuron, Volume *84*

Supplemental Information

Activity-Dependent Gating of Calcium Spikes by A-type K+ Channels Controls Climbing Fiber Signaling in Purkinje Cell Dendrites

Yo Otsu, Païkan Marcaggi, Anne Feltz, Philippe Isope, Mihaly Kollo, Zoltan Nusser, Benjamin Mathieu, Masanobu Kano, Mika Tsujita, Kenji Sakimura, and Stéphane Dieudonné

SUPPORTING INFORMATION

Activity-dependent gating of calcium spikes by A-type potassium channels in Purkinje cell dendrites controls climbing fiber signaling at parallel fiber spines.

Yo Otsu, Païkan Marcaggi, Anne Feltz, Philippe Isope, Mihaly Kollo, Zoltan Nusser, Benjamin Mathieu, Masanobu Kano, Mika Tsujita, Kenji Sakimura and Stéphane Dieudonné*.

- **Corresponding author: dieudon@biologie.ens.fr**

TABLE OF CONTENTS

Supplementary figures on line

Figure S1: Quantitative mapping of calcium transients decrement at high frame rate. *Related to Figure 1*... **2**
Figure S2. DHPG application does not modify the somatic complex spike shape. *Related to Figure 2*.. **3**
Figure S3. DHPG-mediated dendritic spike unlocking is not caused by the mGluR1-mediated slow inward current**.** *Related to Figure 3*... **4**
Figure S4. Calcium spikes and sodium spikes are not temporally correlated. *Related to Figure 5*.. **5**
Figure S5. Effect of 4-AP application on the somatic complex spike shape. *Related to Figure 6* **6**
Figure S6. Purkinje cells express a low-threshold inactivating potassium conductance (ISA). *Related to Figure 7*.. **7**
Figure S7. Electron microscopic freeze-fracture replica immunolobeling for Kv4.3 subunit in the cerebellar molecular layer.*Related to Figure 7*.. **8**

Supplementary text on line

Supplemental Experimental Procedures **9**
Evidence for the activation of a slow inward current in the presence of DHPG... **14**
Quantification of the calcium influx mediated by subthreshold signaling and by calcium spike **15**
High-threshold potassium channels limit the regenerative propagation of spikes in the dendrites but do not control dendritic spike unlocking **16**
Supplemental References

SUPPLEMENTAL DATA

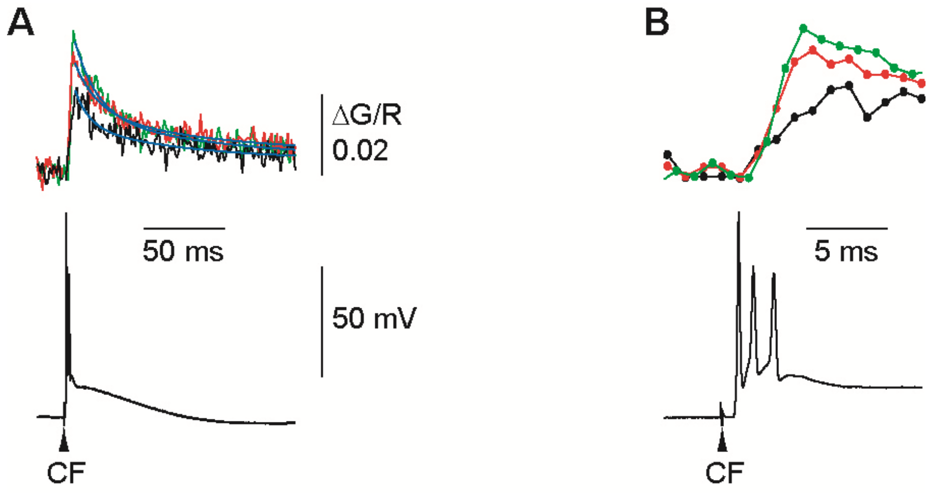

Figure S1: Quantitative mapping of calcium transients at high frame rate. Related to Figure 1

(A-B) Fluorescence transients (top) evoked by CF stimulations were recorded quasi simultaneously at multiple POIs at a frame rate 0.9 kHz while the complex spike (bottom) was recorded at the soma. CFCTs rise was resolved temporally in smooth dendrites (black), in spiny dendritic shafts (red) and spines (green). CFCTs recorded at 0.8 – 1.4 kHz displayed a stereotypical bi-exponential decay (blue lines). Spine: τfast = 11.4 +/- 1.0 ms, τslow = 144.0 +/- 19.5 ms, fast component = 69.2 +/- 2.9 %; Spiny branchlet: τfast = 14.0 +/- 1.3 ms, τslow = 203.5 +/- 18.7 ms, fast component = 65.8 +/- 3.0 %; Smooth dendrite: τfast = 13.6 +/- 2.0 ms, τslow = 312.3 +/- 29.3 ms, fast component = 64.8 +/- 2.4 % (+/- s.e.m.) (n=12 cells; in two of 12 cells the slow component of the decay was too small to be appropriately fitted).

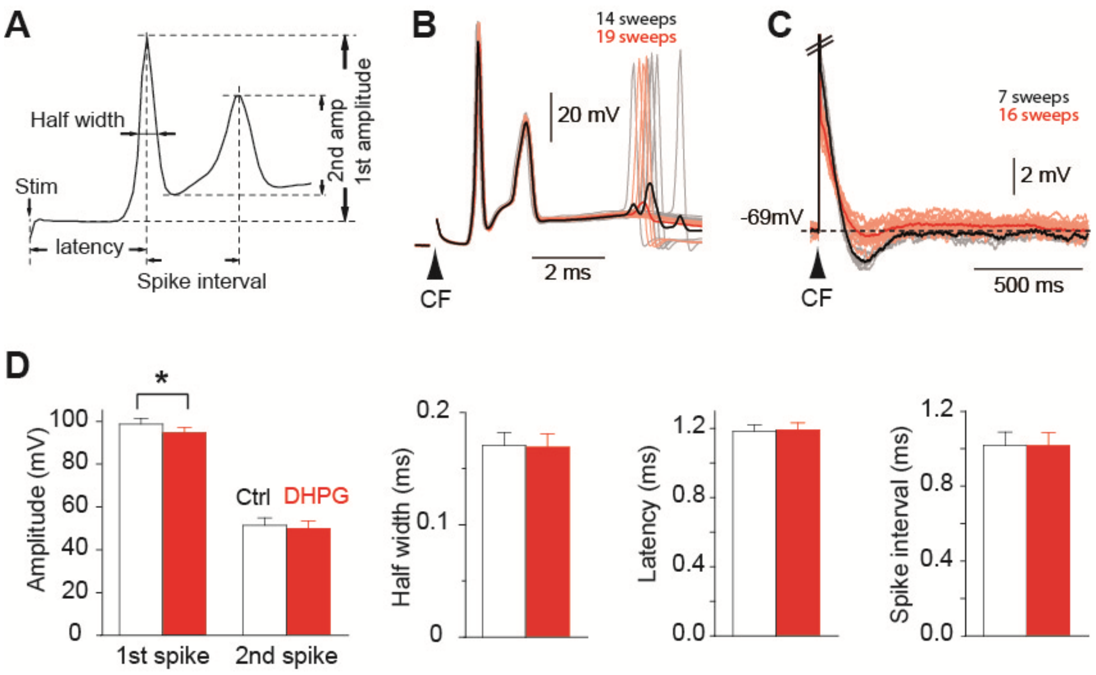

Figure S2. DHPG application does not modify the somatic complex spike shape. Related to Figure 2. (A) Cartoon of the various parameters measured to quantify the complex spike shape before and after application of DHPG in Purkinje cells held at hyperpolarized membrane potentials. (B) Examples of complex spikes recorded in the same cell before (black) and after DHPG application (red). Thick lines indicate averaged sweeps. (C) Enlarged scale in (B). Note that spikes are cut. To compare the afterhyperpolarization followed the complex spikes, the sweeps which had a 3rd sodium spike in the complex spikes were eliminated. See Supplemental Text. Thick lines indicate averaged sweeps. (D) Summary of the measurements (*: p<0.05; 6 cells). The amplitude of the first sodium spikes slightly decreased (control: 98.8 +/- 2.7 mV, DHPG: 94.7+/- 2.6 mV, n=7, p=0.03) while the second sodium spikes remained unchanged (51.7 +/- 3.6 mV vs. 50.0 +/- 3.5 mV, p = 0.18). Neither of the half width of the first sodium spikes (170.3 +/- 11.3 µs vs. 168.9 +/- 11.9 µs, p = 0.27), the first spike latency (1.19 +/- 0.04 ms vs. 1.19 +/ 0.04 ms, p = 0.60) nor the first to second spike interval (1.02 +/- 0.07 ms vs. 1.02 +/ 0.07 ms, p = 0.60), which are indicators of the CF synaptic conductance amplitude (Hansel and Linden, 2000; Schmolesky et al., 2002), were changed. Hence the potentiation of CSCTs by mGluR1 appears to be caused directly by the enhancement of postsynaptic voltage-gated calcium influx and not to a direct potentiation of the CF-EPSP

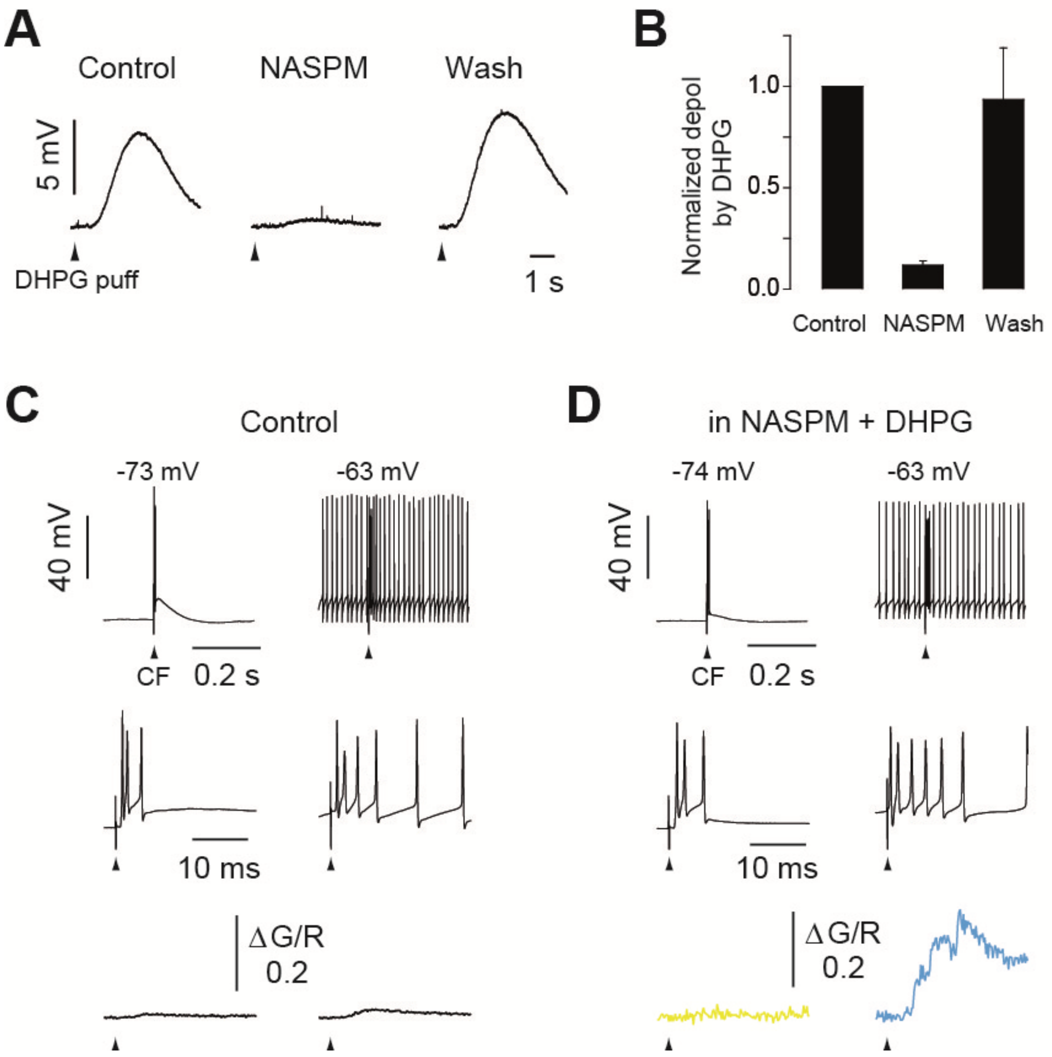

Figure S3. DHPG-mediated dendritic spike unlocking is not caused by the mGluR1-mediated slow inward current. Related to Figure 3.
(A) PC depolarization evoked by puffed DHPG is abolished by NASPM. 100 µM DHPG puffed over the PC dendritic tree for 80ms induces a transient depolarization of 5.8 ± 1.8 mV (peak at 2.4 ± 0.1 s) following the beginning of the puff (n=4). This depolarization is strongly reduced after 3 min superfusion with 100 µM NASPM. The effect of NASPM is reversible after 5 min. (B) On average, NASPM reduced the DHPG puff evoked depolarization to 11.7 ± 2.3 % ($p = 4\ 10^{-5}$; n = 4). (C-D) NASPM did not affect the DHPG-induced spike unlocking (traces from the same cell as in (A)). In control condition (C), the CSCT is barely increased by somatic depolarization through the patch pipette. The protocol is repeated after 5 min superfusion with 100 µM NASPM, and in the presence of 20 µM DHPG (D). Depolarization through the patch pipette then reveals calcium spike unlocking identical to that observed in the absence of NASPM (Figure 3). The averaged CSCT in control condition (firing PC) was 0.029 ± 0.013 (ΔG/R; n = 4), while the averaged CSCT in NASPM and DHPG (firing PC) was 0.24 ± 0.03 (ΔG/R; n = 4; p = 0.001).

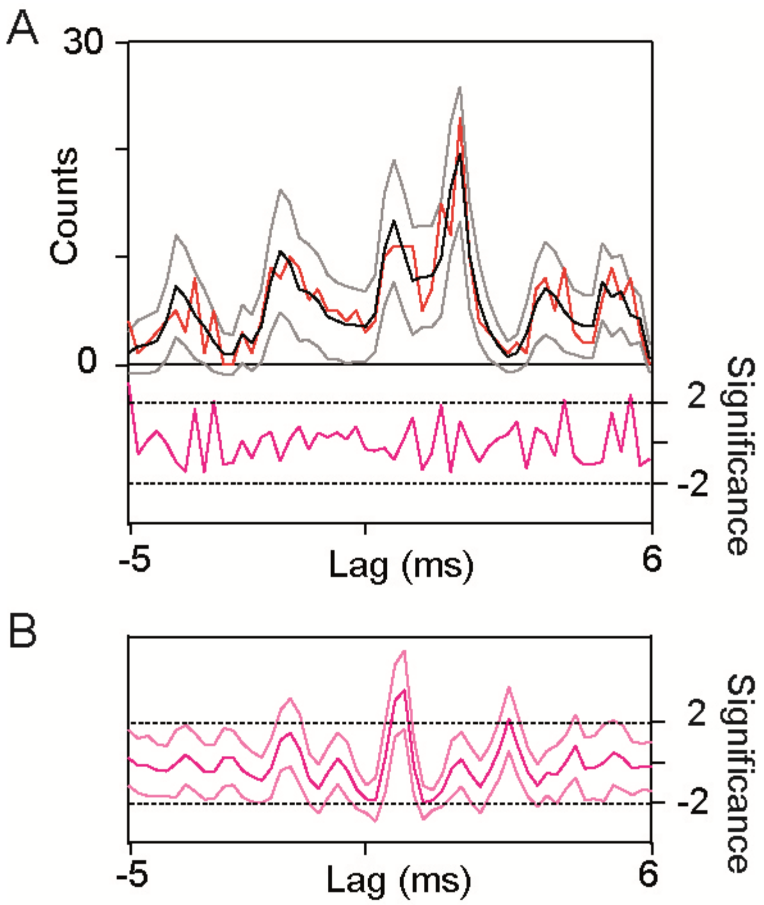

Figure S4. Calcium spikes and sodium spikes are not temporally correlated. Related to Figure 5

The time of occurrence of the peak of somatic sodium spikes and of the half-rise of the dendritic calcium transients were measured for successive climbing fiber stimulations in the presence of DHPG. (A) Red trace: cross-correlogram of the calcium and sodium spikes detected in one Purkinje cell (100 episodes, 0.2 ms bins). All calcium spikes were then shuffled between episodes corresponding to each climbing fiber stimulations and shuffled cross-correlograms were calculated (1000 iterations). Black trace: average shuffled cross-correlogram. Grey traces: +/- 2 S.D. for each point of the shuffled cross-correlogram. Pink trace: significance of the difference between the experimental correlogram and the averaged shuffled correlogram expressed in S.D. of the shuffled correlograms. Note that calcium spikes are not temporally correlated to sodium spikes more than what would occur at random, given the stereotypy of the complex spike bursts. (B) We then verified that true temporal correlation could be detected with a good level of significance. Artificially correlated sets of data, in which each sodium spike was followed by a calcium spike with a Gaussian distribution of latencies, were generated (200 iterations). The mean latency was set to 0.8 ms and the S.D. to 0.2 ms, similar to the temporal precision of the calcium spikes detection, as assessed from paired recordings. The analysis described in **A** was repeated for each set of artificially correlated calcium spikes and the significance relative to the correlation of shuffled traces was calculated. Pink traces represent the average significance ± 2 S.D. and indicate that in most cases a true correlation could be detected with a significance greater than 2 over the random occurrence, at least for the two points surrounding the expected average latency.

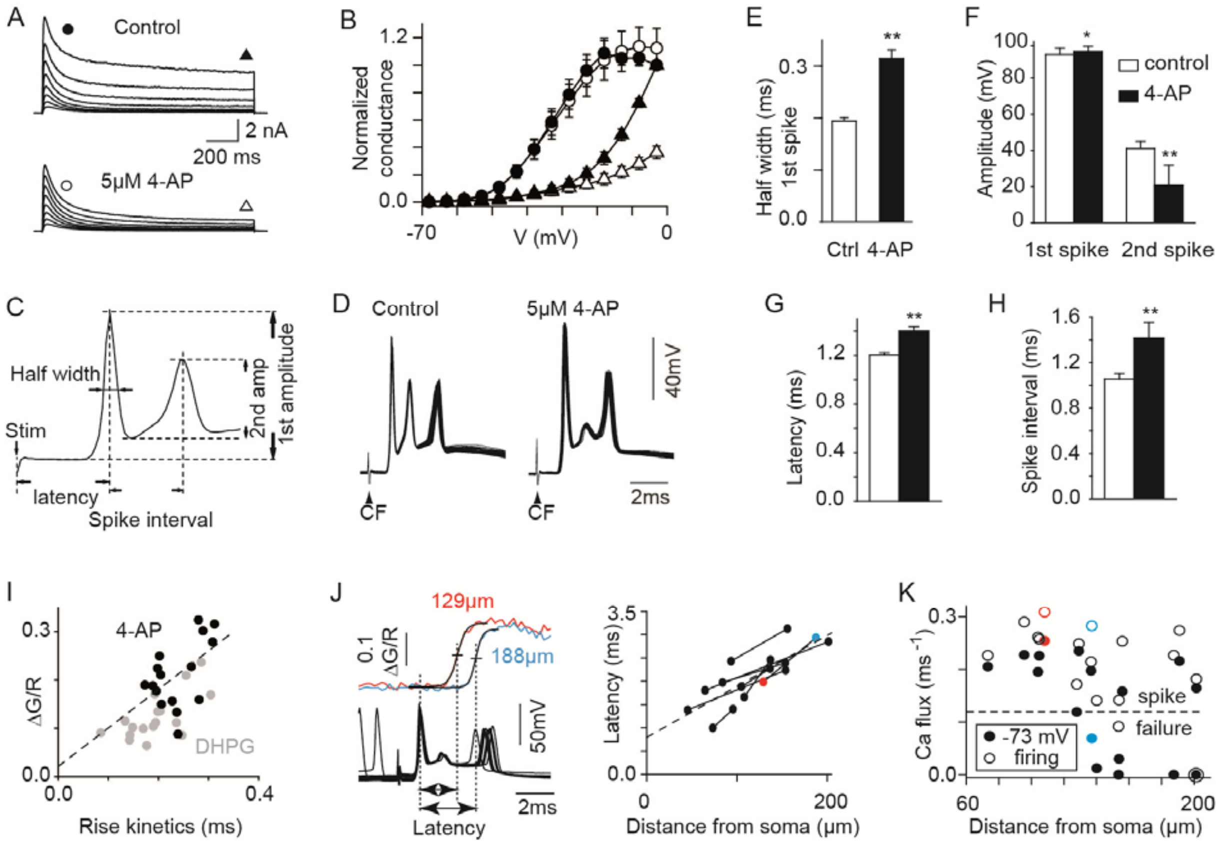

Figure S5 - Otsu et al.

Figure S5. High-threshold delayed rectifier K+ conductance repolarize somatic sodium spikes and dendritic calcium spikes but do not mediate the mGluR1 effect on spike unlocking. Related to Figure 6.

(A) Total (*upper traces*) and 5 µM 4-AP-insensitive (*lower traces*) calcium independent K^+ currents evoked from a holding potential of -73 mV by 1 s depolarizing steps to voltages between -38 and -3 mV in 5 mV increments.

(B) G-V relationship of transient (*circles*) and sustained (*triangles*) K^+ currents averaged from 4 Purkinje cells (postnatal days 5-6). Transient currents mean the difference between peak and sustained currents. *Filled* and *open symbols* indicate the conductance before and after 4-AP application, respectively. These results were obtained by normalizing the conductance at -3 mV before 4-AP application. The transient conductance activation curve is fitted by a Boltzmann function (control vs. 4-AP: $V1/2$ = -33.8 ± 2.0 mV vs. -32.1 ± 1.0 mV, p = 0.465; k = 5.9 ± 0.5 mV vs. 7.1 ± 0.2 mV, p = 0.144; $Gmax$ = 87.5 ± 22.8 nS vs. 94.5 ± 26.8 nS, p = 0.465). Error bar shows ± s.e.m.

(C) Cartoon of the various parameters measured to quantify the complex spike shape in Purkinje cells held at hyperpolarized membrane potentials.

(D) Examples of complex spikes recorded in the same cell before (48 sweeps, *left*) and after (33 sweeps, *right*) 4-AP application.

(E-H) Summary of the measurements (* : p<0.05; ** : p<0.01; 8 cells).

(E) The half width of the 1st sodium spike is increased by 4-AP.

(F) The amplitude of the second sodium spike of the CS was decreased from 44.4 ± 2.6 mV to 17.8 ± 4.3 mV, as expected from increased sodium channels inactivation during the first spike.

(G) The first spike was slightly delayed after the electrical stimulation.

(H) The second spike peaked at longer latency after the first spike (from 1.07 ± 0.06 ms to 1.42 ± 0.14 ms, $p = 0.012$).
(I) Relationship between the amplitudes and the rise kinetics of the CFCTs. *Black* and *gray circles* indicate values obtained in the presence of 4-AP and DHPG (see *filled red circles* in Figure 5C), respectively. *Broken line* is a linear regression for 4-AP data.
(J) Comparison of the timing of the CF-evoked calcium spikes induced in a proximal branchlet (*red*) and in a distal branchlet (*blue*) in the presence of 4-AP. See Figure 5F-G.
(K) Relationship between the calcium flux and the distance from soma at two holding potentials in the presence of 4-AP. *Broken line* indicates the criteria for calcium spikes, obtained in Figure 5D. Examples displayed in (I) are color-coded accordingly.

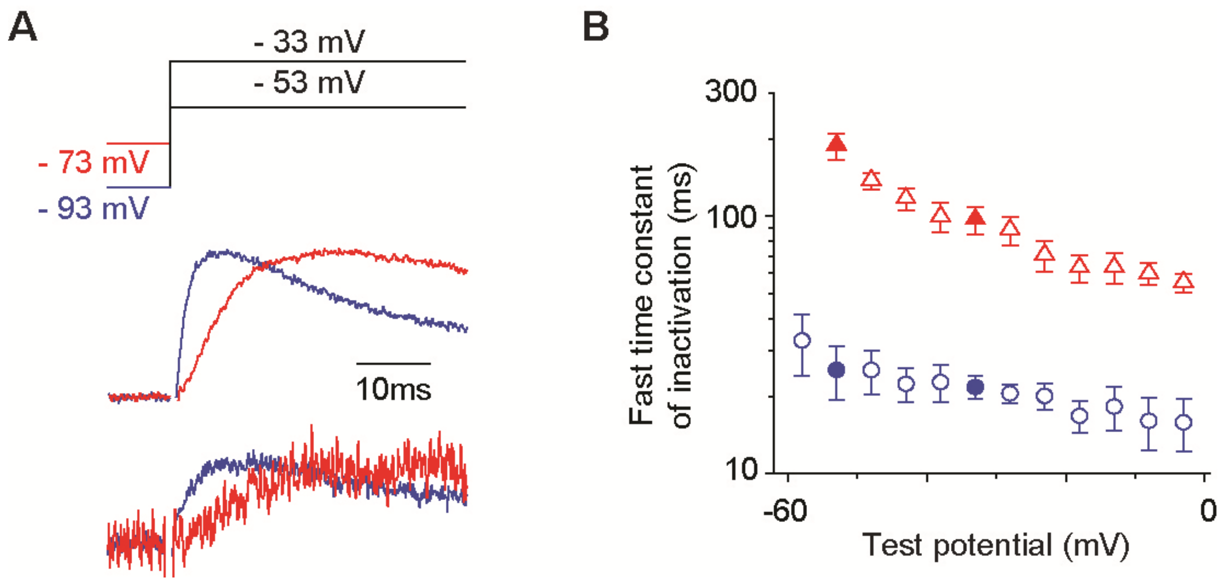

Figure S6. Purkinje cells express a low-threshold inactivating potassium conductance (I_{SA}). Related to Figure 6.

(A) Initial parts of the low threshold I_{SA} current isolated in the presence of TEA and of the medium-threshold A current isolated by inactivation of I_{SA} at -73 mV. Traces were normalized to show the difference in activation and inactivation kinetics.

(B) Fast time constants for the decay phase of K+ currents, when fitted by the sum of two exponential functions. Same protocol as in Fig. 7A with, in *red*, K current evoked at high threshold from a holding potential of -73 mV and in *blue*, K current evoked at low threshold from a holding potential of -93 mV after blockade of the high threshold component by 4 mM TEA. n=7 and 5 respectively. Filled symbols correspond to the recording conditions of the traces displayed in A. Error bars are s.e.m.

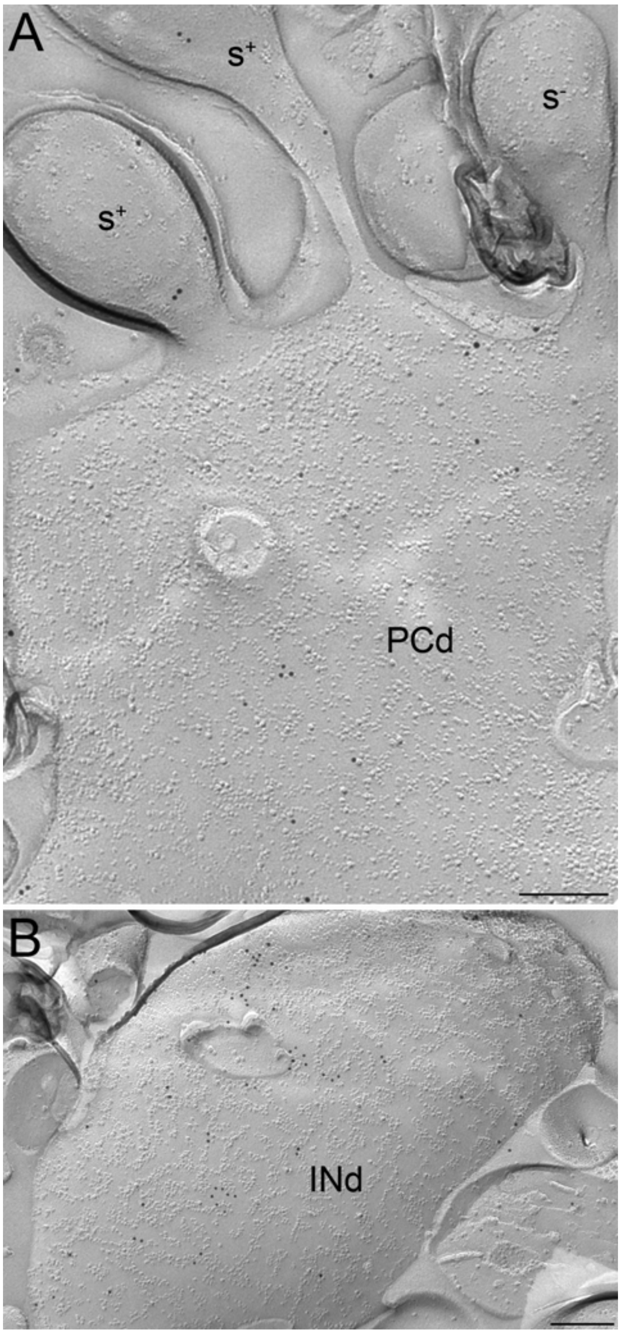

Figure S7. Electron microscopic freeze-fracture replica immunolobeling for Kv4.3 subunit in the cerebellar molecular layer.
Related to Figure 6.
(A) Gold particles labeling the Kv4.3 subunit are located on the P-face of fractured dendritic (PCd) and spine (s+) plasma membranes of a P22 mouse Purkinje cell. The particles are apparently randomly distributed in these membrane areas.
(B) An interneuron dendrite (INd) is strongly immunopositive for the Kv4.3 subunit in the cerebellar molecular layer, consistent with a previous report (Kollo et al., 2006). Scale bars: 0.2 µm. The reactions were carried out according to the method published in Lorincz and Nusser (Science, 2010), using a rabbit anti-Kv4.3 antibody (Chemicon: AB5194).
Cerebellar brain slices

Supplemental Experimental Procedures

Cerebellar brain slices

Parasagittal Slices (250 µm) were prepared from the cerebellum of mice (postnatal day 13-26), according to CNRS animal protocols. Under deep anesthesia with isoflurane, the vermal part of the cerebellum was removed and slices were cut using a vibrating blade microtome (Micron HM 650V) in an ice-cold solution containing (mM) 130 K-gluconate, 15 KCl, 0.05 EGTA, 20 Hepes, 25 glucose and 50 µM D-APV, pH 7.4. Slices were maintained in artificial cerebrospinal fluid (ACSF, 32-34 °C) containing (mM) 125 NaCl, 2.5 KCl, 1.25 NaH2PO4, 26 NaHCO3, 20 glucose, 2 CaCl2, 1 MgCl2 (bubbled with 95% O2, 5% CO2) and then transferred to the recording chamber at 32-34 °C. In some experiments aimed at describing calcium-independent K^+ currents in postnatal day 4-8 mice, slices were kept in ACSF at 32-34 °C for 1 h before being transferred to the recording chamber at room temperature (22-24 °C).

Electrophysiology

Visually-guided patch-clamp recordings were aided by a combination of gradient contrast {Dodt, 2002 #102} and on-line video contrast enhancement. Whole cell recordings in bridge-mode were performed with an Axopatch 2A amplifier or Multiclamp 700B (Axon Instruments). Patch pipettes (resistance 3-4 MΩ) were filled with an intracellular solution containing (mM) 135 KMeSO4 (Fluka), 6 NaCl, 1 MgCl2, 10 Hepes, 10 K2-creatine phosphate (Calbiochem), 4 Mg-ATP, 0.4 Na2-GTP, pH 7.35, and supplemented with a morphological dye (15 µM Alexa 594, Invitrogen) and a calcium-sensitive dye (200 µM Fluo-4 or 500 µM Fluo-5F, Invitrogen) (~300 mOsm). Liquid junction potential was corrected (8 mV). The Purkinje somatic membrane potential is always given as the averaged value in the 550 ms-900 ms window (including sodium spikes) preceding the complex spike. Calcium imaging was started after at least 30 min of whole-cell dialysis. Stimulation electrodes (patch pipettes) were filled with ACSF and put in the granular cell layer to activate the CF and in the vicinity of Purkinje cell dendrites to activate the parallel fiber (PF). The CF inputs to Purkinje cells, identified on the basis of their large all-or-none complex spike, were stimulated every 3-10 s (100-150 µs pulse width). The stimulus intensity for PF inputs was adjusted to induce about 1 mV (0.4-2.3 mV) EPSP from somatic recording in the Purkinje cell at a holding potential of around -75mV. CF/PF-EPSPs were filtered at 3 kHz and sampled 20 kHz. To monitor calcium-independent K^+ currents in Purkinje cells (postnatal day 4-8 mice), voltage-clamp recordings in whole-cell configuration were performed with an Axopatch 200A (Axon

Instruments). Patch pipettes (resistance 3-4 MΩ) were filled with an intracellular solution containing (mM) 138 KCl, 2 MgCl2, 10 Hepes, 10 EGTA, 4 Na2-ATP, 0.4 Na2-GTP, adjusted to pH 7.35 with KOH (295~300 mOsm). The extracellular solution contained 5 mM $MgCl_2$, 0 mM $CaCl_2$, 0.5 mM CsCl, 0.5 μM TTX (Ascent scientific), 0.2 mM $CdCl_2$, 5 μM SR-95531 (Tocris) and 15 mM glucose. The series resistance was less than 10 MΩ and its compensation was set at 85 %. Liquid junction potential was corrected (3 mV). To monitor low threshold A-type K^+ currents (I_{SA}) in physiological conditions (32 °C, postnatal day 7-11 mice), the KMeSO4- base internal solution was used and the standard ACSF was supplemented with 0.5 μM TTX, 5 μM mibefradil, 10 μM ZD7288 (Tocris) and 5 μM SR-95531. The currents were filtered at 5 kHz and sampled 10 kHz. pClamp 9 or 10 (Axon Instruments) software was used for data acquisition.

Calcium imaging and analysis

The analysis was performed with pClamp 9 and 10 (Axon Instruments), Origin 6.1 software (OriginLab) and custom routines in Igor Pro 5.0 (Wavemetrics). The peak of Fluo-4 signals were determined by averaging the signal from 1 point before to 3 points after the Fluo-4 maximum value (~ 4 ms time window around the peak CFCT with a sampling rate of 0.8-1.4 kHz). The peak Fluo-5F signals acquired at high repetition rate was determined by averaging the signal in the raw trace over 10 points (~ 2 ms time window with a sampling rate of 4.5-5 kHz) around the time of maximum Fluo-5F fluorescence in the 9 points box-filtered trace. To detect automatically calcium-spike like events in CFCTs after DHPG application, optical recordings were differentiated using the appropriate kernel and threshold detection was performed on the resulting traces. The peak of each event was determined by averaging the signal from 2-3 point before to 2-3 points after the maximum of the event. The distance from soma or branch points to POIs was measured with NeuronJ {Meijering, 2004 #103} after obtaining raster scan reconstructions of Purkinje cells. To examine the spatial profile of calcium influx across cell population (Figure 1), ΔG/R from each POI was normalized to the averaged values obtained in smooth dendritic regions < 70μm from soma in the same cell.

Drug application

Mibefradil (Sigma), 4-aminopyridine (4-AP; 5 μM) (Sigma) and (S)-3,5-dihydroxyphenylglycine (DHPG; 20 μM) (Tocris) were bath-applied. For mibefradil block, slices were preincubated for 1-2h with mibefradil (2 μM) followed by at least 30 minutes re-equilibration in 1 μM mibefradil solution. For cyclopiazonic acid (CPA) treatment, slices

were perfused for 20 min in the recording chamber or preincubated at least 2 hrs with 25 μM CPA {Galante, 2003 #132}. ω-conotoxin MVIIC (0.5 mM) (Alomone labs) was dissolved in HEPES-buffered solution containing (mM) 141 NaCl, 2.5 KCl, 1.25 NaH_2PO_4, 1.6 $CaCl_2$, 1.5 $MgCl_2$, 10 HEPES (pH 7.4) and locally pressure-applied through a patch pipette (3-4 MΩ) with a Picospritzer II (General Valve co.). To monitor the puff area 50 μM Alexa 594 was added in the puff solution. The pipette was placed near the edge of dendritic arbor of Purkinje cells. Phrixotoxin-2 (1-10 μM) (Alomone labs) {Hirono, 2001 #130} was dissolved in HEPES-buffered solution containing (mM) 126 NaCl, 2.5 KCl, 1.25 NaH_2PO_4, 2 $CaCl_2$, 1 $MgCl_2$, 10 HEPES, 35 D-mannitol, 20 glucose (pH 7.4). Local pressure application (10 μM) with a Picospritzer II (General Valve co.) through a patch pipette was used to monitor effects of the toxin on I_{SA}. The pipette was put close to the slice surface or into the tissue near the Purkinje cell dendrites (postnatal day 9) (Figure 7D and E). For local perfusion of the toxin (1-2 μM), a glass pipette which has larger tip size (~30μm diameter) and longer shaft was used. The toxin was perfused from pial to somatic side 10-30 μm above the slice surface. (Figure 8C and D)

Electron microscopic immunohistochemistry

Four Wistar rats (P43-67) were deeply anesthetized before transcardial perfusion as described previously {Holderith, 2003 #112}. Sagittal sections (60 μm in thickness) were cut from the cerebellar vermis with a vibratome and were washed several times in 0.1 M phosphate buffer. Sections were then blocked in 10% normal goat serum (NGS) in Tris-buffered saline (TBS). After blocking, the sections were incubated in mouse anti-Kv4.3 antibody (Kv4.3-M, K75/41; 1:500; NeuroMab, Davis, CA) diluted in TBS containing 2% NGS and 0.05% Triton X-100. After several washes, a 0.8 nm gold coupled goat anti-mouse antibody was used to visualize the immunoreactions (Aurion, Wageningen, The Netherlands). Ultrasmall gold particles were silver enhanced (EM-SE kit) as described by the manufacturer (Aurion). The specificity of the reaction was evaluated as follows: This mouse monoclonal antibody provided an identical labeling of the cerebellum to those obtained by two additional anti-KV4.3 subunit antibodies, which were directed against different epitopes of the subunit {Kollo, 2006 #72}. The Kv4.3 immunoreactivity was quantified as follows: 22-29 EM micrographs were taken from the molecular layer from each animal. Gold particle densities were measured over the cytoplasm and on a 45 nm wide band at the cytoplasmic side of the plasma membrane {Lorincz, 2002 #111} of Purkinje cells and interneurons. Purkinje cell dendrites were identified based on morphological criteria (presence of lamellar bodies, glial ensheathment, lack of asymmetric

synapses on the dendritic shaft and occasional emergence of spines). Nonspecific labeling densities were measured over Purkinje cell and interneuron nuclei.

Potassium conductances analysis

For K^+ currents, linear leakage and capacitive currents were digitally subtracted by scaling traces at +5 mV command voltages. The amplitude of peak and transient component were calculated by subtracting the basal component before voltage steps and the sustained component which was measured as the mean values of the last 10 ms of the current trace, respectively. The potassium permeability was derived from the *I–V* curve using a modified Goldman–Hodgkin–Katz (GHK) equation of the form: $I\text{k} = G(F^2V/RT)([K^+]_i \exp(FV/RT)-[K^+]_o)/(\exp(FV/RT)-1)$, where G is proportional to the potassium channels permeability, F the Faraday constant, V the membrane voltage, R the gas constant, and T the absolute temperature {Clay, 2009 #129}. Activation conductance curves were determined by fitting a Boltzmann function to G values. The equation used was: $G = G_{max}/\{1 + \exp[(V_{1/2} - V)/k]\}$, where G_{max} is the maximum conductance, $V_{1/2}$ the half activation voltage, and k the slope factor. Inactivation current curves were also fitted with a Boltzmann function, $I\text{k} = I_{max}/\{1 + \exp[-(V_{1/2} - V)/k]\}$. The rising phase and the falling phase in short period from the peak (~ 20ms) were fitted by the product of two exponential terms. The decay phases were fitted with a double- or a monoexponential function.

Statistical analysis

Data are presented as mean ± s.e.m unless otherwise stated. For statistical analyses, Mann-Whitney test, Wilcoxon test, Kruskal-Wallis test, and paired t-test were used as appropriate. Data analysis for electron microscopy was performed using Statistica 6.1 (StatSoft, Inc., Tulsa, OK). Gold densities in the different compartments were compared using repeated-measures analysis of variance (ANOVA) after logarithmic transformation. Tukey HSD test was used as a posthoc test. Values of $P<0.05$ were considered statistically significant.

Supplemental Text

Evidence for the activation of a slow inward current in the presence of DHPG

While the CS shape recorded at hyperpolarized potentials (~ 73 mV) did not vary with bath addition of 20 µM DHPG, the afterhyperpolarization that followed the CS was reduced in amplitude from 2.3 +/- 0.2 mV to 0.9 +/- 0.1 mV (5 cells) and in duration (full width at half maximum) from 101.6 +/- 14.3 to 48.5 +/ 5.9 ms. The time needed for the afterhyperpolarization to reach its peak was reduced from 114.4 +/- 16.7 ms to 88.1 + /- 7.9 ms. The afterhyperpolarization disappeared in 2 cells. Furthermore DHPG application led to the appearance of a slow depolarization (7 cells; amplitude, 0.9 +/- 0.1 mV; duration, 544.8 +/ 102.5 ms peak time, 305.0 +/- 33.7 ms). This slow depolarization could lead to an increased rate of simple spike firing following the CS when the cell was held at more depolarized potentials (frequency ratio of post-CS to pre-CS; control, 100 +/- 0.9 %; DHPG 120 +/- 7 %, n = 6; simple spike frequency preceding the CS was adjusted at 30 - 50 Hz). These data are consistent with previous observations showing the activation of a slow depolarizing metabotropic potential by the CS in the presence of metabotropic agonists (Yuan et al., 2007).

Activation of mGluR1 receptors by a bath-applied agonist (Vranesic et al., 1991) or by synaptic stimulations (Batchelor and Garthwaite, 1997; Dzubay and Otis, 2002) induces a slow inward current in Purkinje cell, which may depolarize the dendrites and increase their excitability. In order to test whether this mGluR1-mediated depolarization is required for dendritic spike unlocking, experiments were performed in 100 µM 1-naphthyl acetyl spermine (NASPM), which has been shown to block the mGluR1-mediated slow inward current (Ady et al., 2014; Canepari et al., 2004). Following 3min of bath-applied 100µM NASPM, the depolarization induced by 80-100ms puff application of 100µM DHPG on Purkinje cell dendrites was nearly abolished (11.7 ± 2.3 % of control; recovery 93.4±25.5% of control; n = 4) (Figure S3A and S3B). However, dendritic unlocking was readily evoked by application of DHPG in the presence of NASPM (n=4 cells; Figure S3C and S3D). The number of dendritic spikes (max 4) was controlled by the somatic depolarization, as in control conditions. We conclude that mGluR1 activation induces voltage dependent unlocking of dendritic calcium spikes by another mechanism than the activation of the mGluR1-mediated slow inward current. .

Quantification of the calcium influx mediated by subthreshold signaling and by calcium spike

Saturation of the dye or of the endogenous calcium buffers could mask the decrement of the calcium transient with distance during suprathreshold signaling. However, in both cases, the redistribution of calcium from the dye to the slow endogenous buffers after the peak of the fluorescence transient should be dramatically decreased. The redistribution of calcium from the dye to the slow endogenous buffers after the peak of the fluorescence transient is measured by the relative amplitude of the fast exponential component of the CSCT decay (Schmidt et al., 2003). It was only slightly reduced by DHPG (control vs. DHPG: *spine*, fast component = 71.1 +/- 3.4 % vs. 54.6 +/- 2.4 %, p=0.04: *spiny branchlet*, fast component = 64.9 +/- 4.1 % vs. 46.6 +/- 3.0 %, p=0.04: *smooth dendrite*, fast component = 64.3 +/- 3.8 % vs. 47.5 +/- 4.3 %, p=0.04, n = 6 cells). Furthermore its time-course remained similar to control, indicating that slower mechanisms like calcium extrusion did not play a significant role (control vs. DHPG: *spine*, τ_{fast} = 13.6 +/- 0.7 ms vs. 14.9 +/- 1.1 ms (p=0.92), τ_{slow} = 189.0 +/- 14.3 ms vs. 167.8 +/- 15.7 ms (p=0.89); *spiny branchlet*, τ_{fast} = 17.4 +/- 1.4 ms vs. 13.7 +/- 1.4 ms (p=0.03), τ_{slow} = 239.1 +/- 24.1 ms vs. 200.0 +/- 20.0 ms (p=0.35); *smooth dendrite*: τ_{fast} = 16.0 +/- 3.9 ms vs. 14.2 +/- 0.9 ms (p=0.92), τ_{slow} = 329.0 +/- 58.9 ms vs. 243.2 +/- 27.5 ms (p=0.35), n = 6 cells; in one of 6 cells under control condition the slow component of the decay was too small to be appropriately fitted) (Figure 2D). These data suggest that saturation of the dye or of the endogenous calcium buffers do not significantly interfere with the linearity of the fluorescence measurements.

The total calcium influx underlying a dendritic spike is equal to the number of dye molecules bound (Higley and Sabatini, 2008), assuming all calcium is bound to the dye at the peak of the fluorescence transient. Measuring the saturated Fluo 4/ Alexa 594 fluorescence ratio allowed us to estimate the fractional saturation of the fluorescence signal, yielding a total calcium influx of about 60 µM per spike. This corresponds to 3.6×10^{-15} C charges for a spine volume of 0.3 μm^3 (Vecellio et al., 2000) and to a peak current of 5 pA. In dendritic shafts, the value found is even higher, as the smaller fluorescence transient is more than compensated by the higher volume to surface ratio. Hence calcium channels themselves are sufficient to produce an overshooting spike in Purkinje cell dendrites.

We measured a sigmoid rise time constant σ of 180 µs for unitary calcium transients. This corresponds to a half-width of the calcium influx of 395 µs. Because calcium channels are the main inward charge carrier in the dendrites (Stuart and Hausser, 1994), spike-related

calcium influx is the cause of membrane depolarization and may outlast active repolarization by the duration of Cav2.1 channels deactivation. The width of the dendritic calcium spikes depolarization may thus be similar to the width of the somatic sodium spike (200 μs). Selective block of high-threshold Kv3 channels by low concentrations of 4-AP exerts similar effects on somatic sodium spike duration and dendritic calcium transient duration (Figure 6), confirming that the time course of the optical transient reflects the time course of calcium influx.

High-threshold potassium channels limit the regenerative propagation of spikes in the dendrites but do not control dendritic spike unlocking

We investigated whether Kv3 could limit the propagation of spikes in Purkinje dendrites. In young Purkinje cells (P5-6 days), bath application of 5 μM 4-aminopyridine (4-AP) was found to block 63.7 ± 4.6 % (n = 4) of the high-threshold non-inactivating voltage-gated K+ conductances at -3 mV (triangles, Figures S5A and S5B), but left lower-threshold A-type conductances untouched (circles, Figures S5A and S5B). This effect of 4-AP was occluded by 4 mM tetraethylammonium (TEA) (3.5 ± 9.8 %, n = 4), which almost completely blocks Kv3 channels {Coetzee, 1999 #71}. The effect of 4-AP on the features of the complex spike was quantified (Figures S5C to S5H). The width of the first sodium action potential of the complex spike was increased by 4-AP from 195.9 ± 8.5 μs to 314.3 ± 16.8 μs (n = 8, p = 0.012) (Figures S5E), as previously reported for the block of Kv3 channels {Martina, 2007 #38;Raman, 1999 #40}. Sodium channel inactivation was increased leading to a reduced amplitude and increased delay of the following low amplitude spikes (Figures S5F, S5H).

Having established the specificity of a low concentration of 4-AP on high-threshold potassium channels, we examined its effect on calcium signaling in spiny branchlets. 4-AP incubation induced a large potentiation of CFCTs at all recording sites, but one (3.5 ± 0.6 fold, p = 0.001, n=16; Figures 5H and 5I). The rise kinetics of these potentiated transients was similar to that of unitary calcium transients induced by DHPG and linearly correlated with their amplitude (r = 0.52, p = 0.037) (Figure S5I). This single dendritic calcium spike propagated away from the soma at a speed of 91 μm ms-1 (r = 0.82, p = 0.00012, n = 15) (Figure S5J). Because Kv3 channels open at highly depolarized potentials, their blockade is not likely to change spike initiation threshold, but Kv3 blockade would allow the spikelet observed at the onset of the CF EPSP in smooth dendrites to grow into a full-blown propagated calcium spike. Small somatic sodium spikelets of the complex spike were not able to propagate, as multiple dendritic unitary calcium transients were never observed. Overall,

the duration and amplitude of 4-AP-induced spikes were increased by 26 % (0.24 ± 0.01 ms vs. 0.19 ± 0.01 ms, $p = 0.019$) and 75 % (ΔG/R at distances 70 – 210 µm from soma; 0.21 ± 0.02, $n = 16$ vs. 0.12+ / 0.01, $n = 17$, $p < 0.001$), respectively relative to that of DHPG-induced spikes (Figure S5I). The peak calcium flux was increased to 0.22 ± 0.02 ΔG/R.ms-1 ($n = 16$, $p = 0.018$), as broadened calcium spikes will open more calcium channels. Hence Kv3 channels play quantitatively similar roles for the fast repolarization of somatic sodium spikes and dendritic calcium spikes.

While Kv3 channels play a role to prevent the undue initiation of a full-blown calcium spike at the onset of the complex spike, they are clearly not the effectors of the mGluR1 voltage-dependent spike unlocking. Indeed, bursts of calcium spikes were never induced by 4-AP, even at depolarized potentials, contrary to what happens in DHPG. Nevertheless, blocking Kv3 channels with 4-AP can be used to override the regulation of calcium spike initiation in the proximal dendrites and to study whether spike propagation in distal dendrites is regulated in a voltage-dependent manner (Figure 5J and S5K). Hyperpolarization to -73 mV only slightly reduced the spike amplitude in proximal dendrites (< 120 µm from soma) (depolarized/hyperpolarized = 113.5 ± 3.9 %, $n = 5$, $p = 0.043$) (Figures 5J and S5K), but blocked the propagation of spikes induced by 4-AP into distal dendrites (7 failures out of 12 recordings between 120 µm and 200 µm, closed circles, Figure S5K). Upon hyperpolarization, persistence of the proximal spike and distal failure were recorded simultaneously in the same cell (Figures 5J and S5K). Hence, spike propagation in the distal dendrites is regulated by powerful voltage-dependent mechanisms, which can stop calcium spikes even after 4-AP potentiation. Downregulation of this voltage-dependent gating mechanism by mGluR1 over the whole dendritic tree is most likely necessary to ensure full unlocking and distal spike propagation.

Supplemental References

Ady, V., Perroy, J., Tricoire, L., Piochon, C., Dadak, S., Chen, X., Dusart, I., Fagni, L., Lambolez, B., and Levenes, C. (2014). Type 1 metabotropic glutamate receptors (mGlu1) trigger the gating of GluD2 delta glutamate receptors. EMBO reports *15*, 103-109.

Batchelor, A.M., and Garthwaite, J. (1997). Frequency detection and temporally dispersed synaptic signal association through a metabotropic glutamate receptor pathway. Nature *385*, 74-77.

Canepari, M., Auger, C., and Ogden, D. (2004). Ca2+ ion permeability and single-channel properties of the metabotropic slow EPSC of rat Purkinje neurons. J Neurosci *24*, 3563-3573.

Dzubay, J.A., and Otis, T.S. (2002). Climbing fiber activation of metabotropic glutamate receptors on cerebellar purkinje neurons. Neuron *36*, 1159-1167.

Higley, M.J., and Sabatini, B.L. (2008). Calcium signaling in dendrites and spines: practical and functional considerations. Neuron *59*, 902-913.

Kollo, M., Holderith, N.B., and Nusser, Z. (2006). Novel subcellular distribution pattern of A-type K+ channels on neuronal surface. J Neurosci *26*, 2684-2691.

Lorincz, A., and Nusser, Z. (2010). Molecular identity of dendritic voltage-gated sodium channels. Science (New York, NY *328*, 906-909.

Schmidt, H., Stiefel, K.M., Racay, P., Schwaller, B., and Eilers, J. (2003). Mutational analysis of dendritic Ca2+ kinetics in rodent Purkinje cells: role of parvalbumin and calbindin D28k. The Journal of physiology *551*, 13-32.

Stuart, G., and Hausser, M. (1994). Initiation and spread of sodium action potentials in cerebellar Purkinje cells. Neuron *13*, 703-712.

Vecellio, M., Schwaller, B., Meyer, M., Hunziker, W., and Celio, M.R. (2000). Alterations in Purkinje cell spines of calbindin D-28 k and parvalbumin knock-out mice. The European journal of neuroscience *12*, 945-954.

Vranesic, I., Batchelor, A., Gahwiler, B.H., Garthwaite, J., Staub, C., and Knopfel, T. (1991). Trans-ACPD-induced Ca2+ signals in cerebellar Purkinje cells. Neuroreport *2*, 759-762.

Yuan, Q., Qiu, D.L., Weber, J.T., Hansel, C., and Knopfel, T. (2007). Climbing fiber-triggered metabotropic slow potentials enhance dendritic calcium transients and simple spike firing in cerebellar Purkinje cells. Molecular and cellular neurosciences *35*, 596-603.
